# Accelerated organelle movements can indirectly affect the motility of other organelles in plant cells

**DOI:** 10.1242/jcs.264573

**Published:** 2026-09-25

**Authors:** Jin-Mo Gu, Alex Keith, Kylie Smith, Andreas Nebenführ

**Affiliations:** Department of Biochemistry & Cellular and Molecular Biology, University of Tennessee, Knoxville, TN 37996-1939, USA

**Keywords:** Cytoplasmic streaming, Organelle movements, Myosin XI, Actin filaments

## Abstract

Organelle movements in plants are driven by myosin XI motors moving along the actin filament cytoskeleton. Several experiments have suggested that indirect effects such as organelle–organelle interactions or hydrodynamic flow might be responsible for organelle movements. To test these models, we altered the motile behavior of specific organelles in *Nicotiana benthamiana* leaf epidermal cells and examined the effects of these perturbations on other organelles. Recruiting myosins to organelles with ‘Booster’ constructs resulted in significant increases of linear motions, whereas binding organelles to cortical microtubules with ‘Anchor’ constructs lead to diminished movements of the targeted mitochondria, peroxisomes and ER, respectively. We did not observe consistent effects of Anchor constructs on non-targeted organelles. Mitochondrial Boosters increased the motility of all tested organelles, presumably by leading to a reorganization of actin filaments. By contrast, ER Boosters resulted in enhanced movements for only Golgi stacks and peroxisomes, suggesting a specific interaction between these organelles. Collectively, this novel engineering approach to modify the movements of specific organelles demonstrates that organelle motility in plant cells can occur independently but nevertheless is coordinated between different types of organelles.

## Teaser

**Highlighted Article:** Using novel fusion proteins that alter the motility of specific organelles, this research uncovered interactions between some organelle types that support models linking the motile behavior of different organelles.

## INTRODUCTION

Dynamic organelle movements are one of the hallmarks of active cellular processes, such as cell growth, migration and division (Bornens, 2008; Chocano-Coralla and Vidali, 2024; Nebenführ and Dixit, 2018; Tominaga and Ito, 2015). In plant cells, cytoplasmic streaming, the collective movement of cytoplasm including organelles, is a prominent feature that was first described in the late 18th century (Corti, 1774). Use of specific inhibitors has revealed that cytoplasmic streaming depends on an intact actin cytoskeleton (Shimmen and Yokota, 1994). More recently, research in *Arabidopsis thaliana* has demonstrated that organellar movements and cytoplasmic streaming are mainly mediated by myosin XI motor proteins moving along the actin cytoskeleton (reviewed in Nebenführ and Dixit, 2018). Knockout of single or several myosin XI genes compromises multiple organelle movements and resulted in reduced cell growth (Avisar et al., 2008; Madison et al., 2015; Ojangu et al., 2007, 2012; Park and Nebenführ, 2013; Peremyslov et al., 2015, 2008, 2010; Prokhnevsky et al., 2008; Ueda et al., 2010).

Although this research clearly establishes the dependence of organelle movements on myosin activity, it is not able to unequivocally resolve the mechanism(s) by which myosin motors propel organelles in plant cells. By analogy with the structurally related myosin V motors in animals and fungi, it has been assumed that each myosin XI motor binds to the surface of an organelle and pulls it along an actin filament (Li and Nebenführ, 2008b; Shimmen and Yokota, 2004; Tominaga and Ito, 2015). In support of this model, myosin XI dimers have been shown to take processive steps along actin filaments *in vitro* (Tominaga et al., 2003) whereas their C-terminal globular tail domain is able to bind to organelles (Li and Nebenführ, 2007, 2008a). Intracellular localization of GFP-labeled myosin tail constructs in plant cells further supports the idea that myosin XI directly binds to organelles (Avisar et al., 2009; Li and Nebenführ, 2007; Sattarzadeh et al., 2011). For example, myosin XI-I has been shown to bind to the transmembrane proteins WIT1 and WIT2 on the outer nuclear envelope to drive nuclear migration (Tamura et al., 2013). Similarly, binding of myosin XIK to MRF7, a member of the MyoB family containing the DUF593 domain (Kurth et al., 2017; Peremyslov et al., 2013), has been found to recruit this motor to Golgi stacks (Perico et al., 2021). Taken together, these results support the model of active organelle transport by direct binding of myosin XI motors to adaptor proteins on their surface.

Other results, however, are not easily compatible with this direct-binding model. For example, single myosin XI knockout mutations typically lead to reduced speeds of several different organelle types (Liu et al., 2024; Madison et al., 2015; Peremyslov et al., 2008; Tian et al., 2021; Wang et al., 2020; Yang et al., 2023) and this effect is enhanced when several myosin genes are mutated (Liu et al., 2023; Peremyslov et al., 2010; Prokhnevsky et al., 2008; Ueda et al., 2010). These findings suggest redundancy among the different myosins, although localization data for tail constructs indicate that different isoforms target different organelle types. In addition, fluorescently labeled myosin XI tail constructs can reduce the movement of several organelle types, even in cases where the labeled tail is not associated with the organelles exhibiting a slowdown (Avisar et al., 2012, 2009; Sparkes et al., 2008), suggesting an indirect effect of the dominant-negative constructs.

To explain these discrepancies, two alternative modes of organelle motility have been proposed (reviewed in Geitmann and Nebenführ, 2015). In the first model, organelles that do not carry their own motors associate with another, actively moving organelle. This concept of indirect-active movement, also referred to as ‘hitchhiking’ (Christensen and Reck-Peterson, 2022), resulted from the observation that altered ER structure in *rhd3* mutants coincided with reduced ER dynamics and diminished motility of Golgi stacks, mitochondria and peroxisomes (Stefano et al., 2014). This model is consistent with the realization that many organelles in eukaryotic cells maintain intimate contacts and are often tightly associated with each other (Perico and Sparkes, 2018; Sparkes et al., 2009a; Staehelin, 1997; Valm et al., 2017).

The second model assumes that active movement of a subset of organelles generates a hydrodynamic flow in which other organelles can be moved along passively (Houtman et al., 2007). This model is supported by the observation that the heterologous expression of the μNS protein from a mammalian reovirus in plant cells leads to inclusion bodies that show movement behaviors very similar to those of endogenous organelles (Peremyslov et al., 2015). The nature of the actively moving organelles generating the hydrodynamic flow is not known, although both the ER (Ueda et al., 2010) and small vesicles (Peremyslov et al., 2015) have been proposed.

These three motility models make distinct predictions for the behavior of organelles. For example, active movement by myosins on the organelle surface can be expected to be independent of the behavior of other organelles, while indirect or passive movements should be influenced by the motility state of other organelles that directly interact with motor proteins. Similarly, perturbations of organelles driving hydrodynamic flow can be expected to affect most if not all other organelles, while the indirect-active model would predict that only organelles that physically interact with the actively moving organelle should be affected by changes in its motility. The goal of the current study was to test whether these indirect effects can be detected and quantified by utilizing a novel approach designed to perturb the motility of individual organelle types.

Several approaches have been employed in mammalian cells to alter the motility of specific organelles. For example, knocking out or overexpressing syntaphilin, an anchor protein of neuronal mitochondria, successfully modified mitochondria motility (Kang et al., 2008). Optogenetic approaches to modulate organelle motility by inducing light-responsive binding between kinesin or dynein motor proteins and RAB11 vesicles have demonstrated that proper positioning of these recycling endosomes is crucial for axon growth (van Bergeijk et al., 2015). To allow for more flexibility, we designed modular ‘Booster’ and ‘Anchor’ constructs to perturb the motility of specific organelles. Specifically, Boosters were designed to generate a gain-of-function phenotype by recruiting additional myosin XI motors to the organelle surface, whereas Anchors were expected to lead to a loss-of-function phenotype by attaching organelles to a relatively static structure inside the cell. To evaluate the effects of these Booster and Anchor constructs, we targeted three different organelle types and examined the effects on both targeted and non-targeted organelles with advanced image analysis tools.

## RESULTS

### Altering specific organelle movements with Booster and Anchor constructs

To understand the effect of specific organelle movements on cytoplasmic streaming, we designed novel fusion proteins to selectively change the behavior of individual organelle types. First, we designed ‘Booster’ constructs that should recruit additional myosin motors to specific organelles for active transportation. Similarly, we assembled ‘Anchor’ constructs to trigger binding to cortical microtubules for reduction of organelle motility. We selected peroxisomes, mitochondria and the ER as target organelles as they allowed us to test different predictions. Specifically, plant cells typically contain few peroxisomes but many mitochondria, which allowed us to explore whether organelle number played a role in potential indirect effects. The ER was selected as it is present throughout the cytoplasm and has been proposed to play a dominant role in the indirect-active movement model (Stefano et al., 2014).

The transmembrane domains of PEX12, TOM7 and CNX1 were used to target these Boosters and Anchors to peroxisome, mitochondria and ER membranes, respectively (Fig. 1A), as previous studies have confirmed that their overexpression does not interfere with the functions of the targeted organelles (Allen et al., 2002; Fan et al., 2005; Liu et al., 2016). For Booster constructs, the coding region of each membrane marker was fused to that of the DUF593 domain of MyoB1 from *Arabidopsis thaliana*, a universal adaptor for various myosin XIs (Kurth et al., 2017; Peremyslov et al., 2015; Perico et al., 2021). For Anchor constructs, the same membrane proteins were fused to the microtubule-binding domain of murine MAP4 protein (Marc et al., 1998). All constructs were transiently expressed by *Agrobacterium* infiltration in *Nicotiana benthamiana* epidermal cells (Batoko et al., 2000) together with different organelle markers (Nelson et al., 2007). Initial tests revealed that the Booster and Anchor constructs were only found on all of the targeted organelles (Fig. 1B–D), although they were not always evenly distributed over the surface of the organelles. The mito-Booster constructs often were concentrated in a bright spot on one end of mitochondria, whereas mito-Anchors were more spread out along the length of the organelles (Fig. 1B). By contrast, ER-Boosters were widely distributed along the ER, whereas ER-Anchors were primarily found in a subset of ER elements (Fig. 1D). The Booster constructs were able to recruit myosin motors to the targeted organelle as demonstrated by co-expression of the mitochondrial Booster with the full-length myosin YFP–XIK fusion protein (Park and Nebenführ, 2013) (Fig. S1A,B). Similarly, the Anchor constructs resulted in greater colocalization of the targeted organelle with microtubules (Fig. S1C–H).

**Fig. 1.**
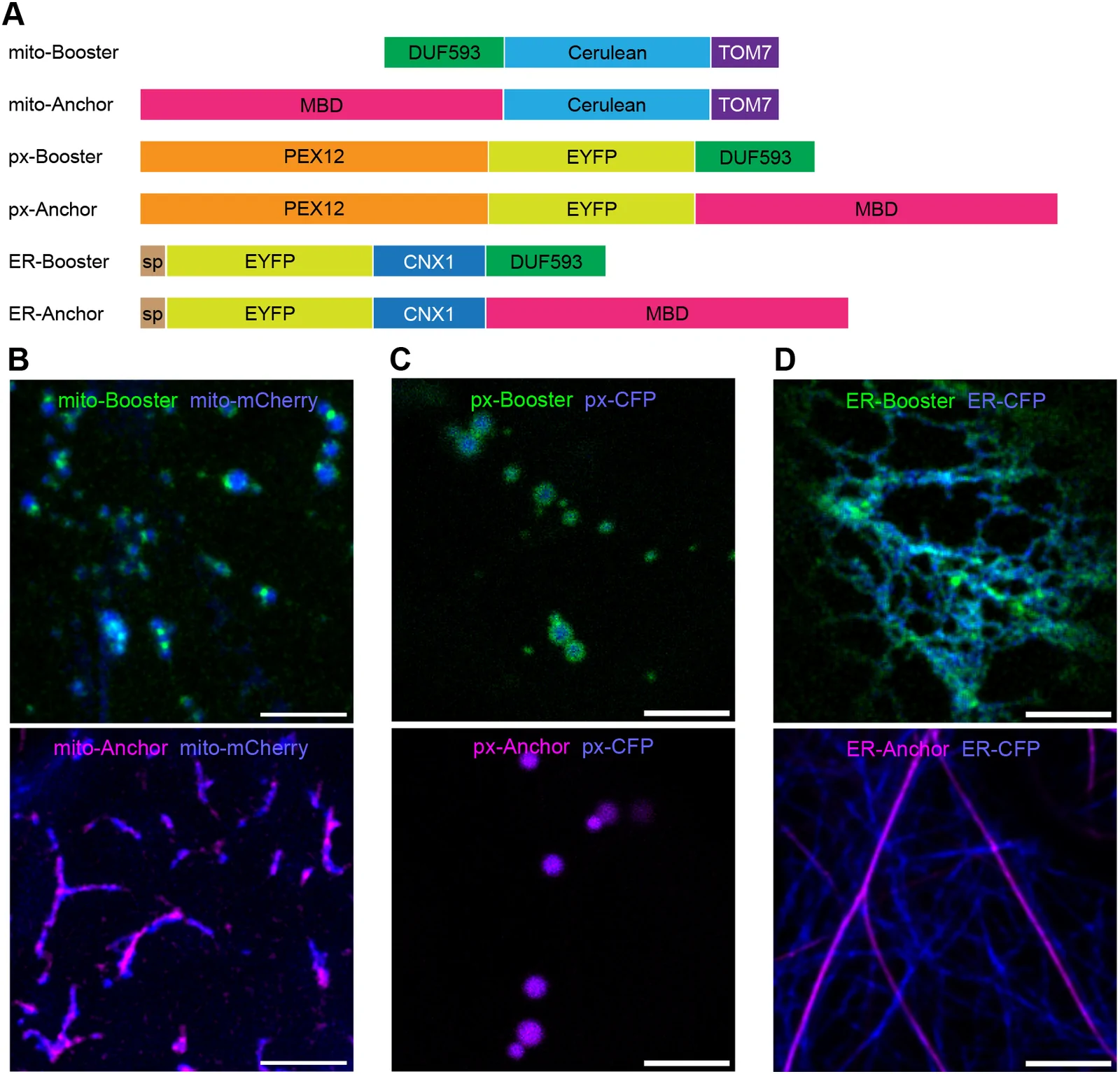
**Booster and Anchor constructs target specific organelles.** (A) Schematic domain organization of Booster and Anchor constructs targeting mitochondria (mito), peroxisomes (px) and the endoplasmic reticulum (ER). Booster constructs contained the DUF 593 myosin-binding domain of MYOSIN BINDING PROTEIN 1 (MYOB1; green), whereas Anchor constructs contained the MDB microtubule-binding domain of mouse microtubule-associated protein 4 (MAP4; hot pink). Organelle targeting was achieved by fusing DUF593 or MDB domains to the transmembrane domain of organelle-specific proteins. For mitochondria, the C-terminal transmembrane domain of TRANSLOCON OF THE OUTER MITOCHONDRIAL MEMBRANE 7 (TOM7; purple) was used. Peroxisomes targeting was achieved with the transmembrane domains of PEROXIN-12 (PEX12; orange). The single transmembrane domain of CALNEXIN1 (CNX1; blue) targeted the ER; note that the N-terminal fusion of EYFP places the tag into the lumen of the ER. In this case, the signal peptide (sp, brown) of WAK2 was added to the N-terminus to ensure insertion into the ER membrane. The constructs included a fluorescent tag (Cerulean for mitochondria, EYFP for peroxisomes and ER) to enable visualization of the fusion proteins. (B) Localization of mito-Booster (green) and mito-Anchor (magenta) constructs to mitochondria labeled with mCherry (mt-rb, Nelson et al., 2007; blue). To detect Booster and Anchor constructs, the standard Cerulean marker was replaced with a double-EYFP tag. Note that the mito-Booster construct often accumulated on one side of the mitochondria, whereas the mito-Anchor construct was found along the length of the elongated mitochondria. (C) Localization of px-Booster (green) and px-Anchor (magenta) constructs to peroxisomes labeled with CFP (px-cb, Nelson et al., 2007; blue). In some cells, the px-Booster also labeled small objects that did not have a clear peroxisome-CFP signal. (D) Localization of ER-Booster (green) and ER-Anchor (magenta) constructs to the ER labeled with CFP (er-cb, Nelson et al., 2007; blue). Note that the ER-Booster construct is broadly, although not evenly, distributed over the ER surface. The ER-Anchor construct is primarily found on a subset of more-or-less straight ER elements. Images representative of eight to ten individual cells. Scale bars: 5 μm.

To capture organelle movements, we performed timelapse imaging with organelle markers in the presence or absence of the different Booster and Anchor constructs. Movement analysis of punctate organelles (mitochondria, peroxisomes and Golgi stacks) followed an established pipeline that filters out linear movements that were interpreted as directional steps along actin filaments, whereas small-scale ‘wiggling’ movements that resembled Brownian motion were considered likely independent of myosin motors. Consecutive linear steps were labeled as runs, whereas the non-directional wiggling motions were characterized as pauses and assumed to represent random diffusion (Nebenführ, 2020). ER speed measurements were performed with optical flow methods that measure displacements of fluorescence fluctuations along individual ER elements in the AnalyzER software (Pain et al., 2019), which further distinguished between narrow ER tubules and wider ER cisternae. In addition, we collected persistency data, which reflects how static individual ER elements are (Sparkes et al., 2009b). For details, see the Materials and Methods.

### Mitochondrial movements are effectively engineered by mitochondria-targeted Boosters and Anchors

As a first test of the efficacy of Booster and Anchor constructs, we expressed a mitochondrial marker (mt-yb, Nelson et al., 2007) with or without the corresponding mitochondrial Booster (mito-Booster) or Anchor (mito-Anchor) in *N. benthamiana* leaves. The mito-Booster construct resulted in more elongated linear trajectories compared to that seen in control cells which showed relatively sporadic and random patterns (compare Fig. 2B with A; also see Movie 1). By contrast, the mito-Anchor construct suppressed mitochondrial motility dramatically as most organelles remained at the same location during the observation period and showed only few extended trajectories (Fig. 2C; Movie 1).

**Fig. 2.**
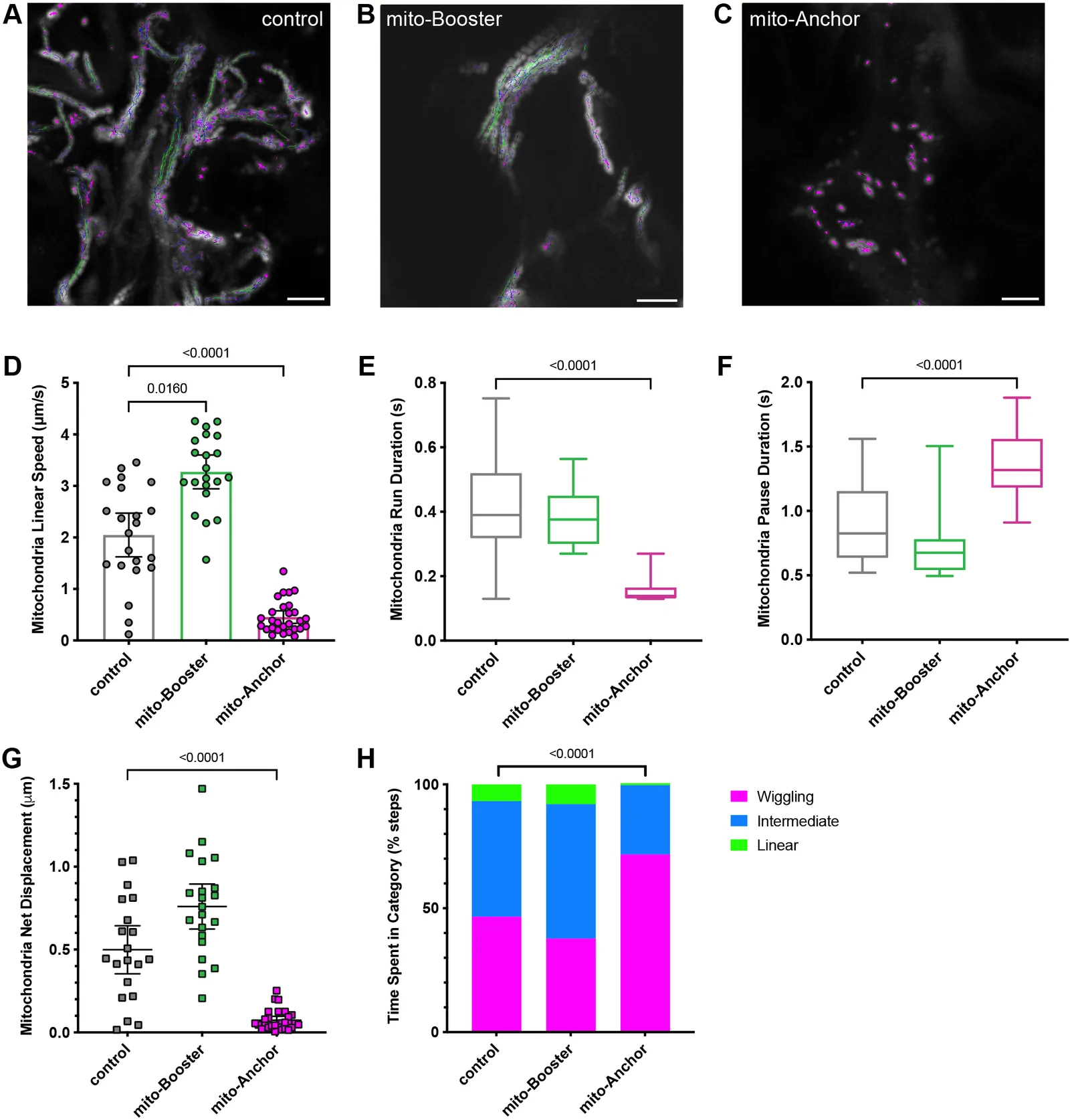
**Mito-Boosters increase mitochondrial movements while mito-Anchors inhibit these movements.** (A–C) Tracking of mitochondria in *N. benthamiana* leaf epidermal control cells (A), cells expressing mito-Boosters (B) or cells expressing mito-Anchors (C). Green tracks represent linear movements whereas magenta tracks represent wiggling motions; blue tracks are intermediate between the two. Individual mitochondria were tracked for 200 frames. Tracks are superimposed on a maximal intensity projection of all frames. Representative cells displaying average linear speed and motility patterns. Scale bars: 5 μm. (D) Linear speed of mitochondria increased with expression of mito-Boosters and decreased with expression of mito-Anchors. (E) Mito-Anchors reduced the duration of continuous linear movements (‘runs’). (F) Mito-Anchors increased the duration of continuous wiggling motions (‘pauses’). (G) Mitochondria did not move as far during run episodes when mito-Anchors were expressed. (H) Expression of mito-Anchors shifted mitochondrial movements from linear to wiggling. Stacked bars indicate percentage of tracking steps assigned to linear (green), intermediate (blue) or wiggling (magenta) motions for control, mito-Booster and mito-Anchor cells. Significant differences in proportions to control cells are indicated (chi-square test). (D,G) Every dot represents the upper quartile of values from one cell. Error bars represent 95% confidence interval around mean. Significant *P*-values relative to control cells are indicated (Kruskal–Wallis test with Dunn correction). (E,F) Box indicates interquartile range with median of upper quartile duration values; whiskers indicate minimal and maximal values. Significant P-values relative to control cells are indicated (Kruskal–Wallis test with Dunn correction). Sample sizes were: *n*=22 cells for control, 21 cells for mito-Booster and 28 cells for mito-Anchor conditions.

Quantitative analysis of the movement data revealed that linear speeds showed a significant 60% increase with the Booster construct and a 78% decrease with Anchor constructs (Fig. 2D; Table S1). Interestingly, wiggling speeds of mitochondria were also increased by mito-Booster constructs (Fig. S2A), suggesting that these seemingly random movements also can depend, at least in part, on myosin activity. To quantify the processivity of mitochondrial movements, we measured the duration of run episodes and found these to be significantly reduced in the presence of mito-Anchors whereas mito-Boosters did not affect the duration of runs (Fig. 2E), possibly due to the limited observation time and area. The mito-Anchor constructs also extended the pause durations of mitochondria by more than 50% (Fig. 2F), suggesting that it was more difficult for mitochondria to exit a wiggling state when bound to microtubules. These changes in motile behavior resulted in changes in net displacements during runs by both mito-Booster (∼1.5-fold increase) and mito-Anchors constructs (∼8-fold decrease) (Fig. 2G). Although the relative change in net displacements with mito-Booster expression was similar to that of linear speed, it did not reach the level of significance due to the large variation between cells. The reduced run duration and increased pause duration in cells expressing the mito-Anchor construct resulted in a drastic shift in the time spent in the different movement categories (Fig. 2H). In the presence of the mito-Anchor construct, mitochondria spent almost 75% of their time in wiggling mode while linear movements were nearly eliminated. By contrast, the mito-Booster construct had only a mild effect on these parameters (Fig. 2H). Taken together, these results demonstrate that the mito-Booster and mito-Anchor constructs are able to alter the motile behavior of mitochondria in the predicted ways.

Unexpectedly, we observed changes in mitochondrial morphology with the mito-Anchor from small, mostly circular shapes to larger, elongated ones (Fig. S2B), which resulted in significant changes in circularity (Fig. S2C), as if mitochondria were stretched out along microtubules (also compare Fig. 1B). As mitochondrial morphology and their functional integrity are coupled (Mishra and Chan, 2016; Quintana-Cabrera and Scorrano, 2023; Wai and Langer, 2016), we examined the membrane potential changes in mitochondria by using MitoTracker Red CMXRos (Pendergrass et al., 2004). The ratio of mean intensity of MitoTracker to the mitochondria marker mito-YFP was significantly decreased with the mito-Anchor expression (Fig. S2D,E). These data could suggest that forced relocation of mitochondria to the cortical microtubules leads to deterioration of both their morphological homeostasis and their functional integrity.

### Peroxisome-targeted Boosters and Anchors alter peroxisome movements

Next, we examined the peroxisome Booster and Anchor effects on their targeted organelle. As the default motility of peroxisomes was relatively low in pavement cells of *N. benthamiana* leaves under our conditions (Fig. 3A), strongly enhanced peroxisome movements were observed by px-Booster constructs (Fig. 3B) whereas the px-Anchor construct appeared to have limited effects (Fig. 3C; Movie 2). Quantitatively, a more than two-fold increase in linear speeds could be measured in cells expressing px-Boosters (Fig. 3D), which was accompanied by a similar increase in run durations (Fig. 3E) and a 20% reduction in pause durations (Fig. 3F). The combined effect of faster speeds and longer runs was a more than four-fold increase in net displacements during runs in the presence of px-Booster constructs (Fig. 3G). The longer runs and shorter pauses observed with px-Boosters also resulted in a striking shift in motile behavior of the peroxisomes by strongly increasing the time spent in linear motion and reducing the likelihood of non-directed wiggling motions (Fig. 3H).

**Fig. 3.**
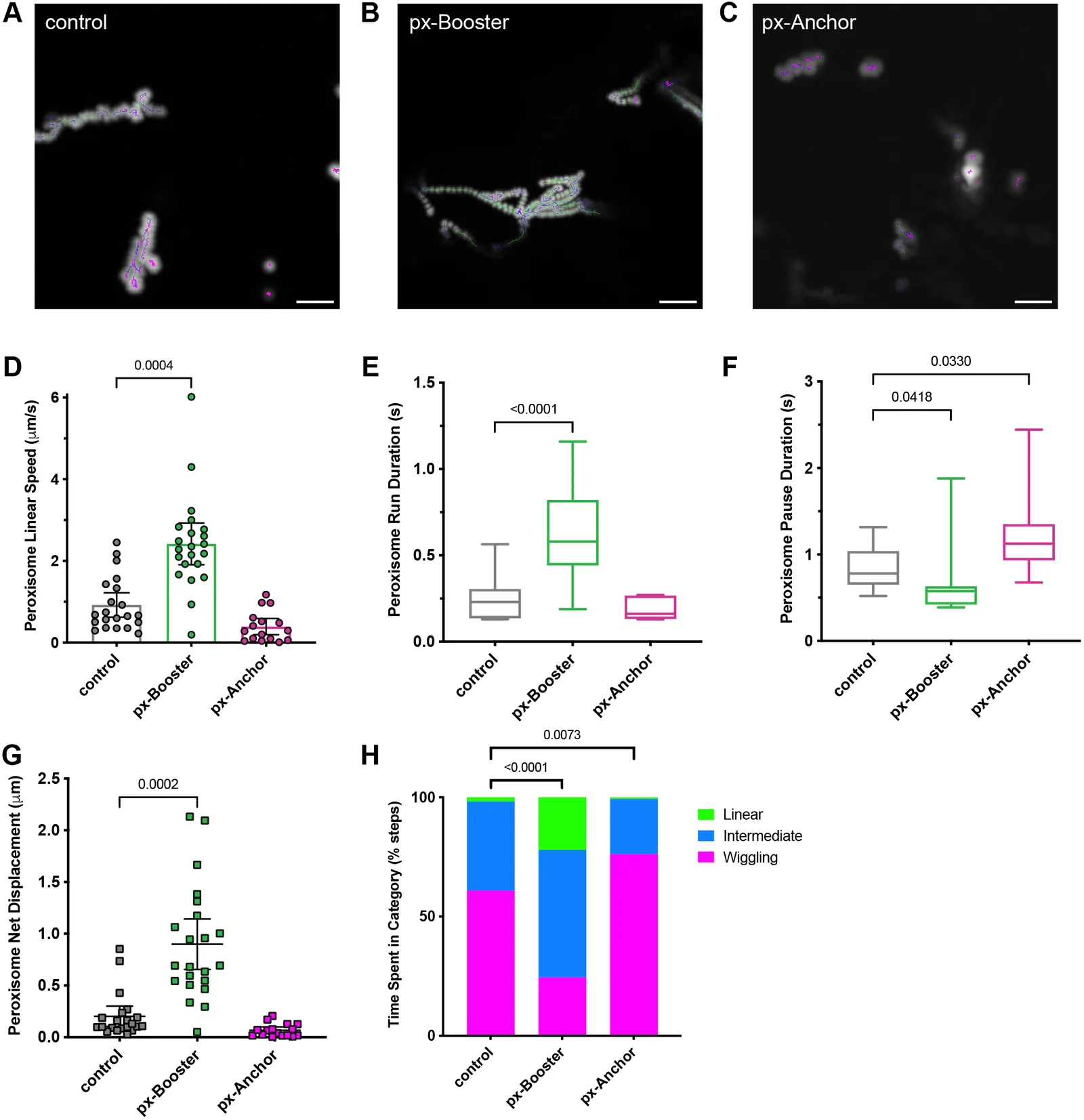
**Px-Boosters increase peroxisome movements while px-Anchors inhibit these movements.** (A–C) Tracking of peroxisomes in *N. benthamiana* leaf epidermal control cells (A), cells expressing px-Boosters (B) or cells expressing px-Anchors (C). Green tracks represent linear movements while magenta tracks represent wiggling motions; blue tracks are intermediate between the two. Individual peroxisomes were tracked for 200 frames. Tracks are superimposed on a maximal intensity projection of all frames. Representative cells displaying average linear speed and motility patterns. Scale bars: 5 μm. (D) Linear speed of peroxisomes increased with expression of px-Boosters. The decrease in linear speeds after px-Anchor expression was not significant. (E) Px-Boosters increased the duration of continuous linear movements (‘runs’). (F) Px-Boosters decreased and px-Anchors increased the duration of continuous wiggling motions (‘pauses’). (G) Peroxisomes moved further during run episodes when px-Boosters were expressed. (H) Expression of px-Boosters and px-Anchors shifted the balance of linear and wiggling movements of peroxisomes. Stacked bars indicate percentage of tracking steps assigned to linear (green), intermediate (blue) or wiggling (magenta) motions for control, mito-Booster and mito-Anchor cells. Significant differences in proportions to control cells are indicated (chi-square test). (D,G) Every dot represents the upper quartile of values from one cell. Error bars represent 95% confidence interval around mean. Significant *P*-values relative to control cells are indicated (Kruskal–Wallis test with Dunn correction). (E,F) Box indicates interquartile range with median of upper quartile duration values; whiskers indicate minimal and maximal values. Significant *P*-values relative to control cells are indicated (Kruskal–Wallis test with Dunn correction). Sample sizes were: *n*=21 cells for control, 22 cells for px-Booster and 16 cells for px-Anchor conditions.

Despite the relatively confined movements of peroxisomes in control cells, we identified further arrest of peroxisome motility by px-Anchor expression. Specifically, pause durations were extended by almost 50% (Fig. 3F) and the behavior shifted even more to wiggling motions while nearly eliminating linear movements (Fig. 3H). Interestingly, the observed wiggling speed of peroxisomes was reduced by more than 50% (Fig. S3A), whereas linear speeds showed a smaller reduction (40%) that was not statistically significant (Fig. 3C). Collectively, these results suggest that px-Boosters and px-Anchors are effective ways to engineer peroxisome motility.

### Enhanced and reduced ER movements by ER Boosters and Anchors, respectively

In eukaryotes, ER tubules and cisternae form complex polygonal structures that can be labeled with fluorescent proteins carrying an ER retention signal in their lumen (Nelson et al., 2007). Although luminal speeds are widely used to determine ER movement speeds (Pain et al., 2019; Ueda et al., 2010), luminal fluid dynamics could in principle be independent of the enclosing membrane. To test whether the luminal speed can reliably represent membrane speed, YFP–CNX1 without the Booster or Anchor component was co-expressed with CFP–HDEL (Nelson et al., 2007) in the same cells and their speeds were compared. Global speed measurements of luminal CFP and membrane-bound YFP correlated closely and did not differ significantly from the predicted 1:1 ratio (Fig. S4A,B), suggesting that luminal speed measurements can serve as reliable readouts of ER motility.

Expression of ER-Booster constructs resulted in spatially uneven increases in ER speeds (compare Fig. 4A and Fig. 4B; Movie 3), presumably because of the uneven distribution of the Booster constructs in the ER membrane (Fig. 1D). Quantitative analysis of ER speeds by optical flow revealed an overall 1.5- to 1.7-fold increase in tubule and cisterna speeds, respectively (Fig. 4D,E), although this might underestimate the effect of the Booster as it includes areas of the ER network with lower presence of the ER-Booster construct.

**Fig. 4.**
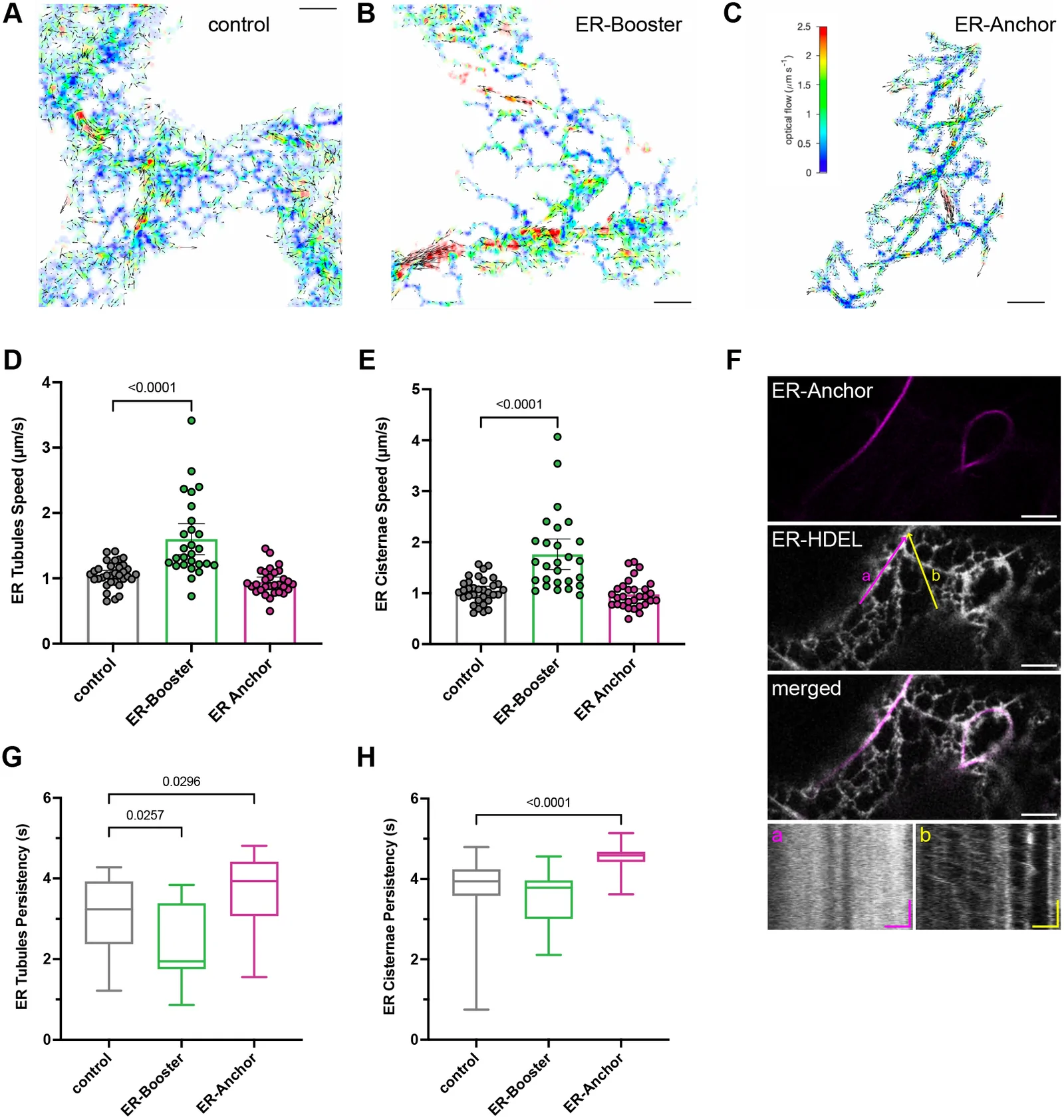
**ER-Boosters lead to faster movements whereas ER-Anchors increase persistency.** (A–C) Optical Flow analysis of ER movements in *N. benthamiana* leaf epidermis in control cells (A), cells expressing ER-Booster (B) or ER-Anchor (C). Color coding of local speeds according to the scale in C indicates local speed of the ER elements. Images show representative frames from video sequence. Scale bars: 5 μm. (D,E) Optical flow speeds of ER tubules (D) and ER cisternae (E) increased with expression of ER-Boosters. Every dot represents the upper quartile of values from one cell. Error bars represent 95% confidence interval around mean. Significant *P*-values relative to control cells are indicated (Kruskal–Wallis test with Dunn correction) (*n*=33 cells for control, 27 cells for ER-Booster and 30 cells for ER-Anchor conditions). (F) ER-Anchor constructs blocked movement of a subset of ER elements. Confocal images showing ER-Anchor (YFP channel, first panel) and the luminal ER marker, ER-HDEL (CFP channel, second panel). Bottom panel a shows kymograph along line a from the second panel; bottom panel b shows kymograph along line b from the second panel. Presence of the ER-Anchor appeared to eliminate movement of ER elements. Scale bars: horizontal, 5 μm; vertical (in kymographs), 2 s. (G) Persistency of ER tubules decreased in ER-Booster cells but increased in ER-Anchor cells. (H) Persistency of ER cisternae increased in ER-Anchor cells. (G,H) Box indicates interquartile range with median of upper quartile values from at least 15 cells; whiskers indicate minimal and maximal values. Significant *P*-values relative to control cells are indicated (Kruskal–Wallis test with Dunn correction) (*n*=32 cells for control, 19 cells for ER-Booster and 25 cells for ER-Anchor conditions).

Comparison of the ER marker (CFP–HDEL) with the YFP signal of the ER-Anchor construct revealed highly uneven distribution of the ER-Anchor in the ER membrane (Fig. 4F; also see Fig. 1D). Regions of the ER that contained the ER-Anchor construct showed very limited motility whereas ER-Anchor-free regions had essentially normal dynamics, as judged by kymographs (Fig. 4F; also see Fig. 4C and Movie 4). As a result of this spatial separation, the overall speeds of ER tubules and cisternae within individual cells were not significantly altered by the ER-Anchor construct (Fig. 4D,E).

Some cells expressing ER-Anchors displayed a drastically altered organization of the anchored ER tubules with the ER-Anchor signal appearing in large, widely curving loops (Fig. S4C). Interestingly, fluorescence signal from microtubules associated with these ER-Anchor tubules was much brighter than that of standard cortical microtubules, suggesting that these represent bundles of microtubules.

In addition to flux of material within ER tubules and cisternae, the rearrangements of individual network elements also define an important aspect of ER dynamics. Persistency of tubules and cisternae can be used to characterize this component of ER dynamics by calculating how long any given pixel within each structure does not move (Griffing et al., 2014; Sparkes et al., 2009b). Here, we captured static pixels up to ∼5 s within each structure and found significant increases of persistency times with ER-Anchor expression (Fig. 4G,H), demonstrating that ER-Anchors can reduce ER structural dynamics in living cells. Conversely, ER-Booster constructs reduced the persistency of ER tubules significantly whereas ER cisternae were only marginally affected by this construct (Fig. 4G,H). These results demonstrate that ER-Boosters and ER-Anchors significantly change the ER dynamics.

### Mitochondrial Boosters increase peroxisome, Golgi and ER movements

After confirming that the Booster and Anchor constructs altered the movements of their targeted organelles in predictable ways, we investigated whether changes in the movements of one organelle would also affect those of other organelles. Peroxisomes in cells expressing the mitochondrial Boosters had shortened pause durations (Fig. 5C), which correlated with more time spent in linear motion and less in wiggling (Fig. S5B). We also noticed small trends to faster linear motions and extended run durations that could not be statistically distinguished from control cells (Fig. 5A,B). These data indicate that enhanced movements of mitochondria have a weak effect on the linear movements of peroxisomes and disrupt their wiggling motions. Importantly, these indirect changes induced by the mito-Booster were smaller than those induced directly by peroxisome Boosters. By contrast, the mitochondrial Anchor had no significant effect on the different movement parameters of peroxisomes (Fig. 5A–C; Fig. S5A,B), which suggests that basal levels of peroxisome movements do not depend on mitochondrial motility.

**Fig. 5.**
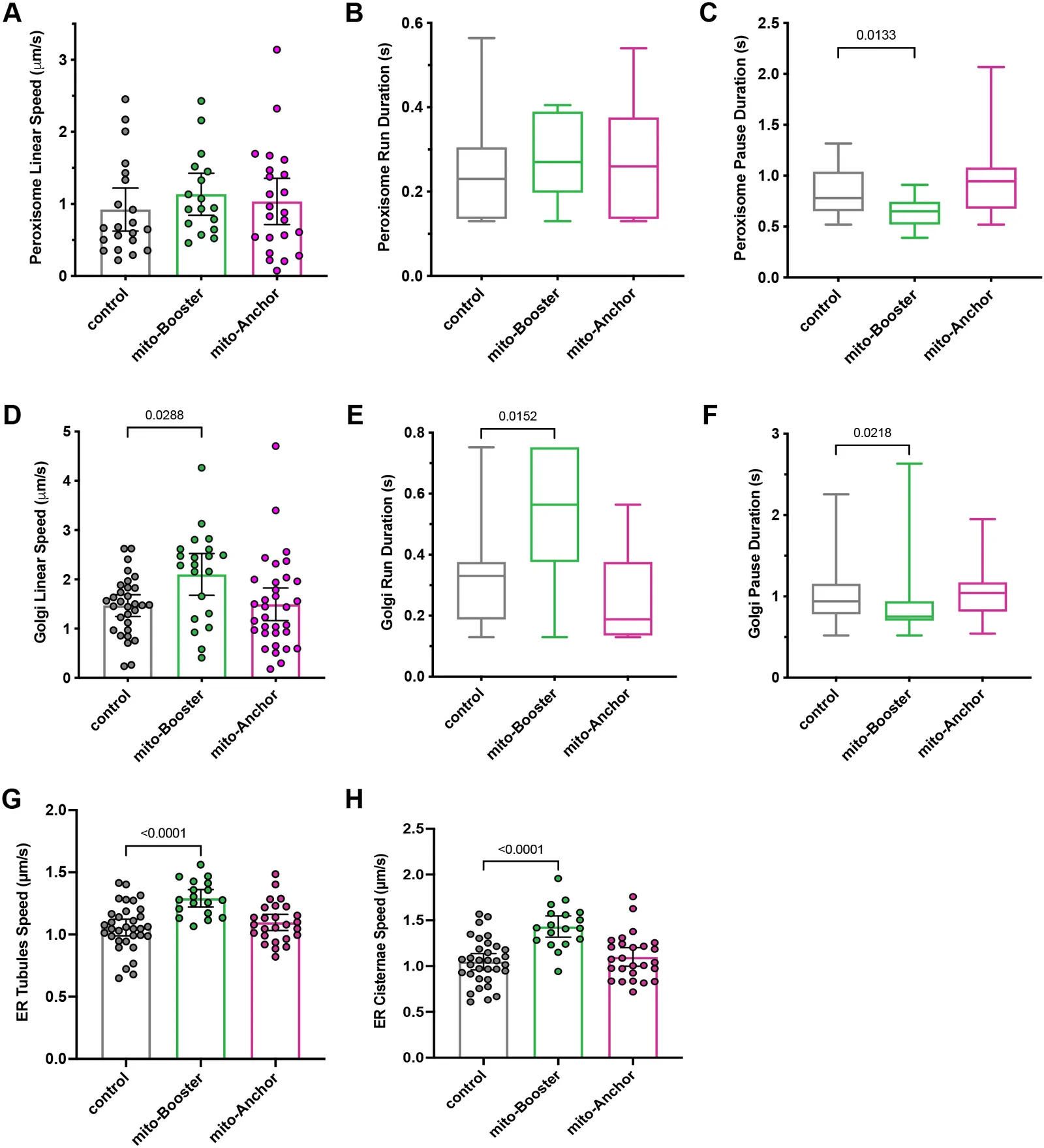
**Mitochondrial Boosters affected movement of several other organelles.** (A) Peroxisome linear speeds (upper quartile) were not significantly affected by mito-Boosters or mito-Anchors (*n*=21 cells for control, 17 cells for mito-Booster and 23 cells for mito-Anchor conditions). (B) Run duration of peroxisomes was not significantly affected by expression of mito-Boosters or mito-Anchors. Box plots show interquartile range, median as well as minimal and maximal values. (C) Pause duration of peroxisomes was reduced by expression of mito-Boosters. (D) Golgi stack linear speeds (upper quartile) increased with the expression of mito-Boosters (*n*=31 cells for control, 21 cells for mito-Booster and 33 cells for mito-Anchor conditions). (E) Run duration of Golgi stacks was strongly increased by expression of mito-Boosters. (F) Pause duration of Golgi stacks was reduced by expression of mito-Boosters. (G) ER tubules moved faster in cells expressing mito-Boosters (*n*=33 cells for control, 18 cells for mito-Booster and 26 cells for mito-Anchor conditions). (H) ER cisternae moved faster in cells expressing mito-Boosters. (A–H) Significant *P*-values relative to control cells are indicated (Kruskal–Wallis test with Dunn correction).

Compared to peroxisomes, boosted mitochondria movements showed more prominent impacts on Golgi stacks. We measured a 1.5-fold increase in linear speed of Golgi stacks cells expressing mito-Boosters (Fig. 5D), which was accompanied by longer run episodes (Fig. 5E) and shorter pauses (Fig. 5F); this resulted in a shift to more time spent in linear and less time in wiggling motion (Fig. S5D). Taken together, these results indicate that enhanced mitochondrial movements can also stimulate Golgi movements by increasing their speed and enhancing their processivity. Interestingly, the mitochondrial Anchor construct did not affect Golgi movements, again suggesting that Golgi movements can occur independently of mitochondria.

Finally, we evaluated ER motility changes upon increased or arrested mitochondrial movements. Global speeds of both ER tubules and cisternae were significantly increased by mitochondrial Booster constructs (Fig. 5G,H), while the persistency of both ER tubules and cisternae was only marginally reduced (Fig. S5E,F). Mitochondrial Anchors, on the other hand, did not exhibit any significant effects on ER dynamics as judged by ER speeds as well as persistency of both tubules and cisternae (Fig. 5G,H; Fig. S5E,F). Overall, these results demonstrate that enhanced mitochondrial motility tends to facilitate translocation of other organelles, whereas suppressed movements of mitochondria have no or only very minor effects on the movements of these other organelles.

### Engineered peroxisome movements do not induce major changes in movements of other organelles

Next, we tested whether the modified movements of peroxisomes could alter the movements of other, non-targeted organelles. Interestingly, we measured a significant drop in run durations of mitochondria in the presence of the px-Booster (Fig. 6B). Other parameters were not affected in a significant way (Fig. 6A,C; Fig. S6A,B), suggesting that the px-Booster construct has limited, and even contradictory, influence on the movement of mitochondria. We similarly found no consistent effects of px-Boosters on Golgi motility (Fig. 6D,E; Fig. S6C,D), except for a drop in pause duration (Fig. 6F). ER dynamics were also not affected by px-Boosters, both in terms of streaming speeds (Fig. 6G,H) and persistency (Fig. S6G,H). In summary, the tested organelles showed only weak changes in their dynamic behavior when peroxisome movements were enhanced by expression of px-Boosters.

**Fig. 6.**
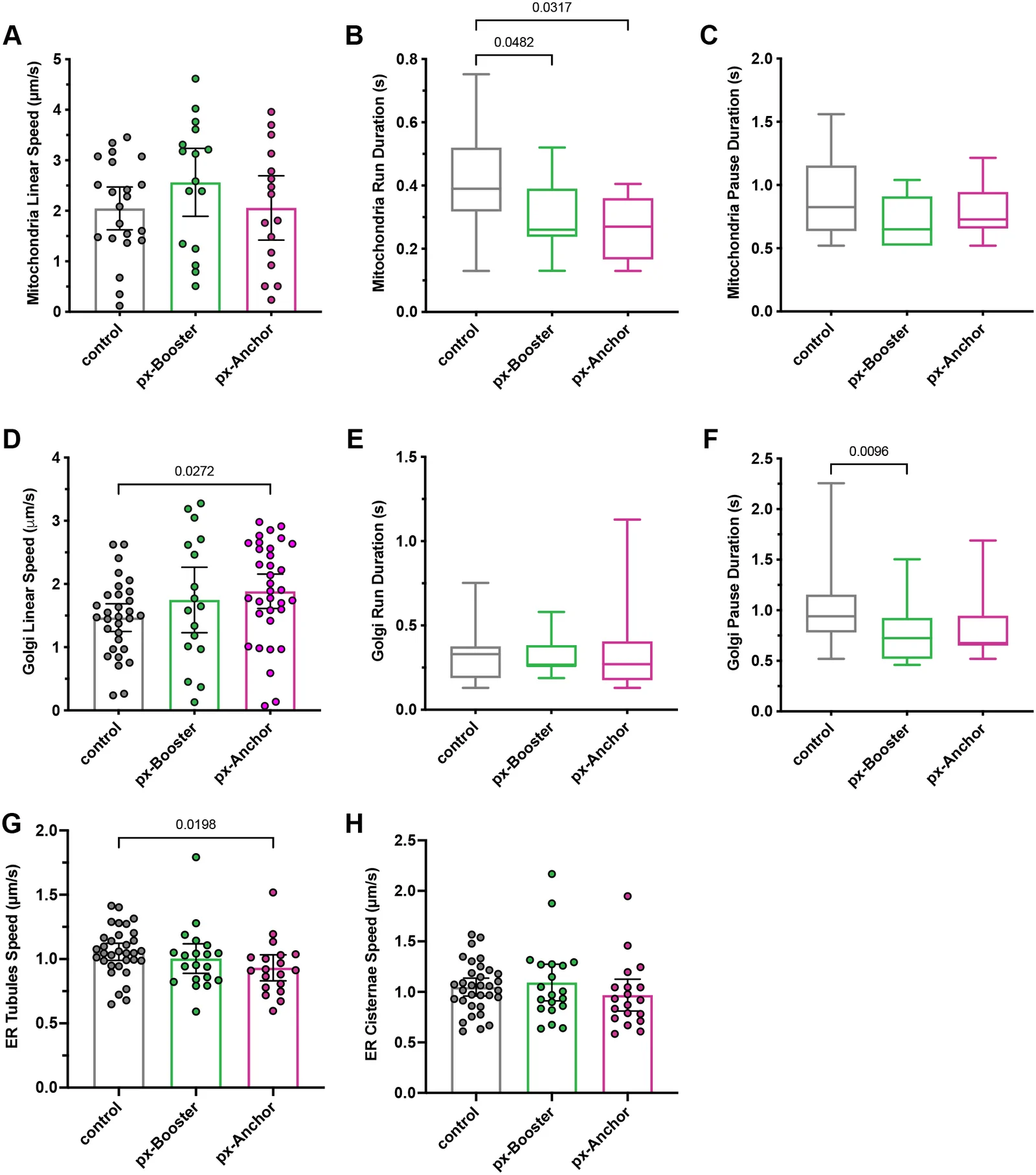
**Changes in peroxisome movements had limited effects on dynamics of other organelles.** (A) Mitochondria linear speeds (upper quartile) were not significantly affected by px-Boosters or px-Anchors (*n*=22 cells for control, 16 cells for px-Booster and 16 cells for px-Anchor conditions). (B) Run duration of mitochondria was reduced by expression of px-Boosters and px-Anchors. Box plots show interquartile range, median as well as minimal and maximal values. (C) Pause duration of mitochondria was not affected by expression of px-Boosters or px-Anchors. (D) Golgi stack linear speeds (upper quartile) increased with the expression of px-Anchors while px-Boosters did not have a significant effect (*n*=31 cells for control, 17 cells for px-Booster and 34 cells for px-Anchor conditions). (E) Run duration of Golgi stacks was not affected by expression of px-Boosters or px-Anchors. (F) Pause duration of Golgi stacks was reduced by expression of px-Boosters. (G) ER tubules moved slightly more slowly in cells expressing px-Anchors (*n*=33 cells for control, 20 cells for px-Booster and 19 cells for px-Anchor conditions). (H) Speed of ER cisternae was not affected by px-Boosters or px-Anchors. (A–H) Significant *P*-values relative to control cells are indicated (Kruskal–Wallis test with Dunn correction).

Similar to the lack of strong effects of the mitochondrial Anchor constructs, the peroxisomal Anchor had minimal effect on the motility of mitochondria, Golgi and ER (Fig. 6; Fig. S6). The only exceptions to this were a drop in run duration for mitochondria (Fig. 6B), a curious and contradictory increase in Golgi linear speed (Fig. 6D), and an unexpected small drop in the speed of ER tubules (Fig. 6G). Taken together, modified peroxisome movements, unlike those of mitochondria, had no systematic impact on modulating the translocation of other organelles.

### ER Boosters increase peroxisome and Golgi linear movements but affect mitochondria only marginally

Given that the ER has multiple interactions with other organelles (Baillie et al., 2020) and has been proposed to drive the motility of associated organelles (Stefano et al., 2014), we tested whether changes in ER dynamics would also affect the motility of other organelles. First, we examined mitochondrial motility in cells with increased or arrested ER movements. Unexpectedly, both ER-Boosters and ER-Anchors did not induce significant changes in linear movement profiles of mitochondria, including linear speeds, run and pause durations, and run net displacements (Fig. 7A–C; Fig. S7A). Curiously, the fraction of time mitochondria spent in linear motion decreased while at the same time non-directional wiggling motions also decreased with expression of the ER-Booster (Fig. S7B). This unusual shift in organelle movements towards ‘intermediate’ behavior might suggest increased short-term fluctuations in stop-and-go behavior of mitochondria.

**Fig. 7.**
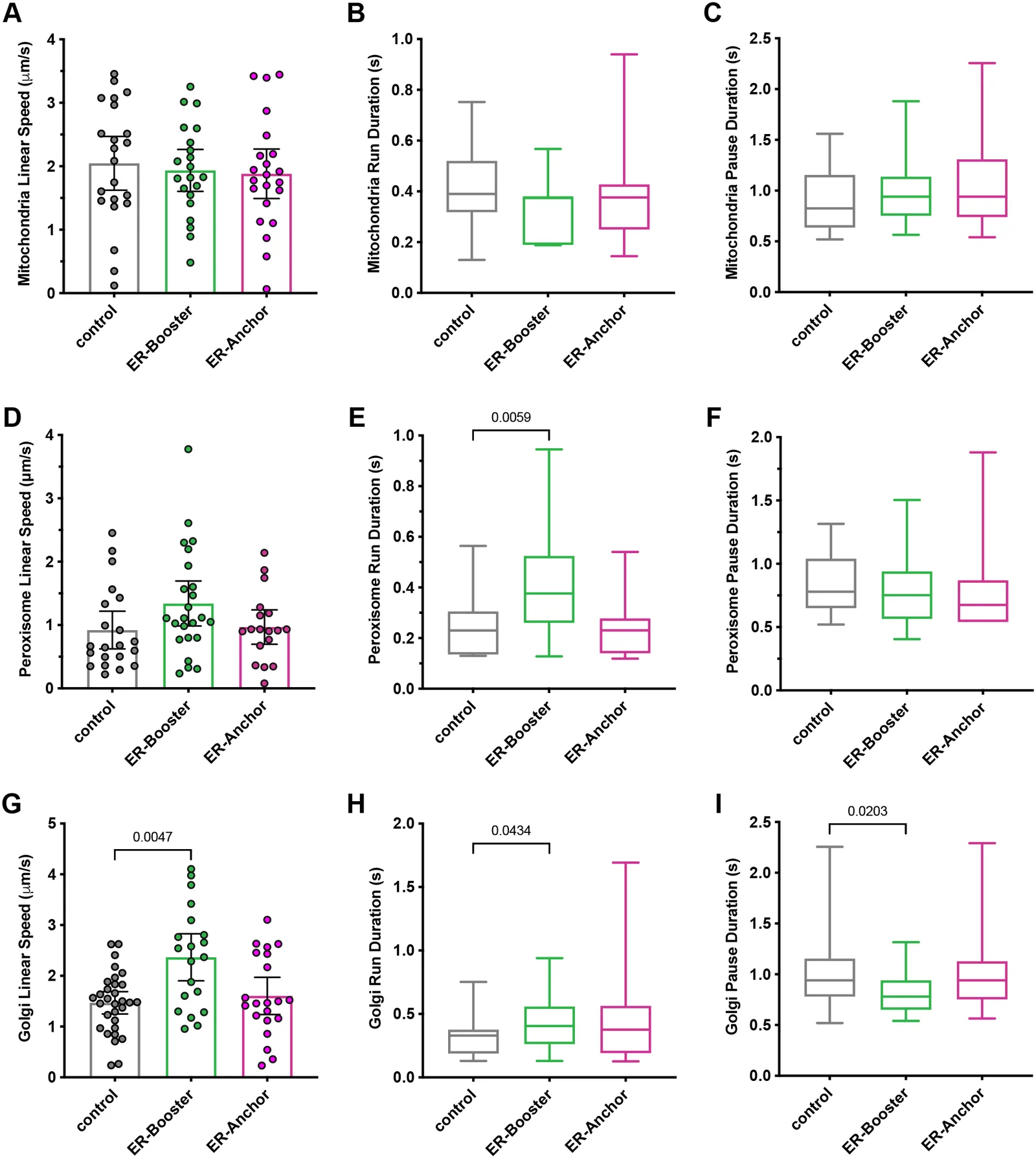
**ER-Booster expression increased the dynamics of some other organelles.** (A) Mitochondria linear speeds (upper quartile) were not significantly affected by ER-Boosters or ER-Anchors (*n*=22 cells for control, 21 cells for ER-Booster and 22 cells for ER-Anchor conditions). (B) Run duration of mitochondria did not change significantly with expression of ER-Boosters and ER-Anchors. Box plots show interquartile range, median as well as minimal and maximal values. (C) Pause duration of mitochondria was not affected by expression of ER-Boosters or ER-Anchors. (D) Peroxisome linear speeds (upper quartile) did not change significantly with the expression of ER-Boosters or ER-Anchors (*n*=21 cells for control, 24 cells for ER-Booster and 18 cells for ER-Anchor conditions). (E) Run duration of peroxisomes was increased by expression of ER-Boosters. (F) Pause duration of peroxisomes was not affected by expression of ER-Boosters. (G) Golgi stack linear speeds (upper quartile) increased with the expression of ER-Boosters (*n*=31 cells for control, 20 cells for ER-Booster and 21 cells for ER-Anchor conditions). (H) Run duration of Golgi stacks was increased by expression of ER-Boosters. (I) Pause duration of Golgi stacks was reduced by expression of ER-Boosters. (A–I) Significant p-values relative to control cells are indicated (Kruskal–Wallis test with Dunn correction).

By contrast, peroxisome motility was increased by enhanced ER movements as demonstrated by longer run durations and run net displacements (∼2.9-fold increase) (Fig. 7E; Fig. S7C). This level of fold increase was even higher than the increase shown with mito-Booster expressions (compare with Fig. 5) and was also reflected in the increased fraction of linear steps and reduction of wiggling steps (Fig. S7D), indicating a strong influence of boosted ER motility on peroxisome movements. ER-Booster expression resulted in only marginally faster peroxisome movements (Fig. 7D) and did not affect pause duration (Fig. 7F).

Finally, Golgi movements increased in linear speeds and in run net displacements (1.64- and 1.82-fold increase, respectively) by ER-Boosters (Fig. 7G; Fig. S7E). While there was only a weak trend in the distribution of movement types towards more linear and fewer wiggling movements (Fig. S7F), we detected significantly extended run and shortened pause durations of Golgi movements with the ER-Booster (Fig. 7H,I). These results demonstrate that enhanced ER movements affected not only the quality but also the quantity of the movements of Golgi stacks.

Interestingly, despite the clear stimulatory effects of ER-Booster constructs on peroxisome and Golgi stack movements, there was no discernible effect of the ER-Anchor construct on these organelles (Fig. 7; Fig. S7).

### Mitochondrial Boosters lead to increased bundling of actin filaments

Augmented movements caused by Booster constructs in some cases revealed various indirect effects in non-targeted organelles, pointing to novel mechanisms that link the movement of some organelles to those of others. Given that loss of myosin activity has been shown to affect actin organization (Madison et al., 2015; Peremyslov et al., 2010; Ueda et al., 2010), we speculated that increased organelle movements in cells expressing Booster constructs might also change the arrangement of actin filaments.

To test this hypothesis, we co-expressed an actin marker with each of the Booster and Anchor constructs and analyzed the structure of the actin network near the plasma membrane with our recently developed actin morphometry pipeline (Akenuwa et al., 2024). Although most of the measured parameters were not different between the treatments, we found that expression of mitochondrial Booster constructs resulted in a significant increase in bundle parameter (Fig. 8A). Control cells showed a dense meshwork of actin filaments with few more prominent filament bundles (Fig. 8B,C). By contrast, we observed increased numbers of long, bright bundles stretching across the surface of the cells expressing the mitochondrial Booster (Fig. 8D–F). Thus, the increased recruitment of myosin XI motors to mitochondria not only caused increased movements of this organelle but also resulted in a reorganization of actin filaments with an increased formation of long straight bundles.

**Fig. 8.**
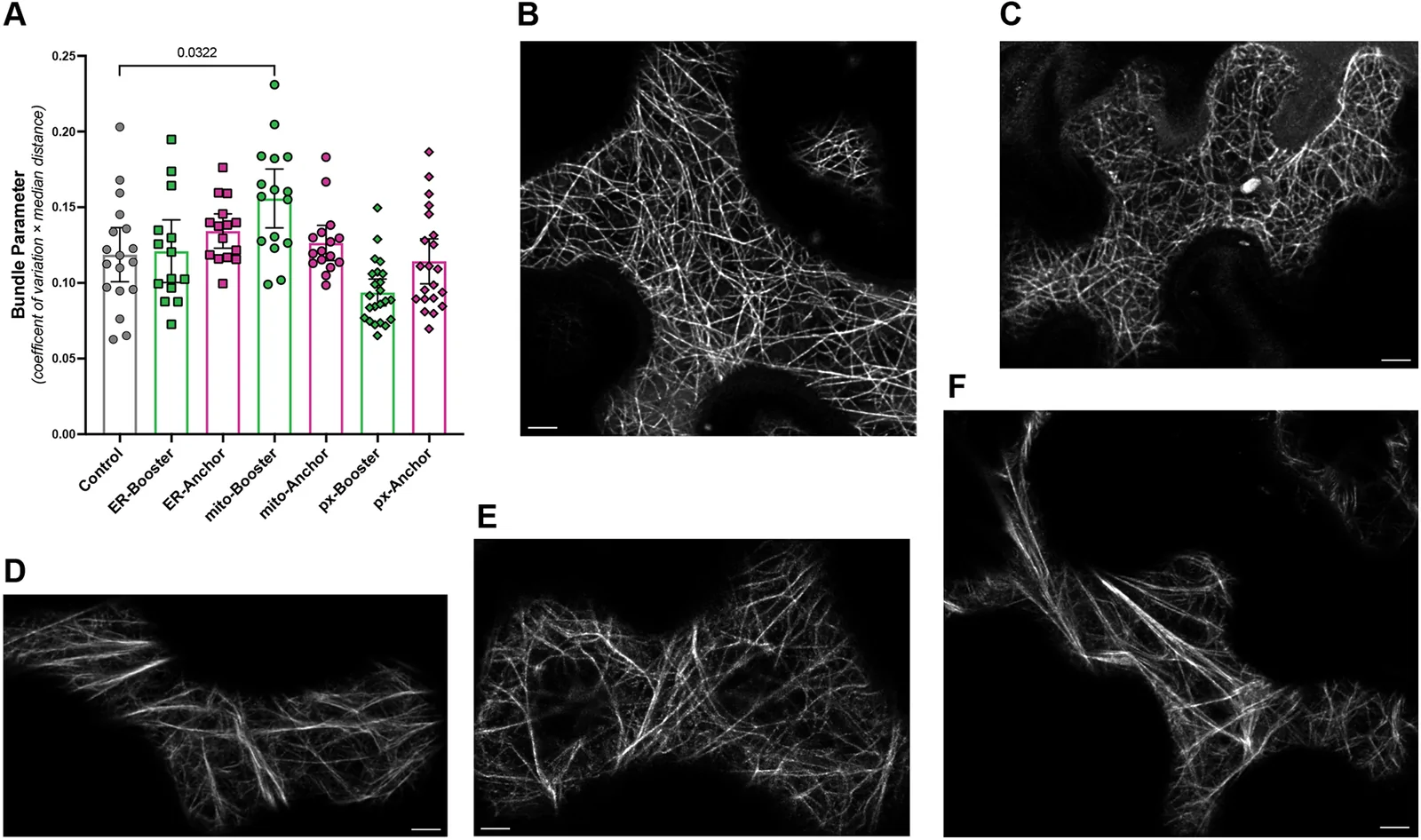
**Mitochondrial Booster constructs lead to increased bundling of actin filaments.** (A) Increased bundling of actin filaments in cells expressing the mito-Booster construct. Every dot represents one cell. Bars show mean±95% confidence interval (Kruskal–Wallis test with Dunn correction) (*n*=15–24). (B–F) Maximal intensity projections of confocal images of actin filaments in the outermost 3 μm of cells expressing YFP–FABD2. Scale bars: 5 μm. (B,C) Actin filament organization in representative control cells. Bundle parameter values were 0.122 and 0.118, respectively. (D–F) Actin filament organization in representative cells expressing the mitochondrial Booster. Bundle parameter values were 0.155, 0.160 and 0.158, respectively.

## DISCUSSION

### Advanced image analysis reveals novel insights into organelle movements

We analyzed organelle movements in plant cells with advanced quantitative image analysis tools that allowed us to demonstrate that different organelles within the same cell type exhibit different movement patterns in terms of linear speed, duration of directed movement episodes and relative time spent moving along actin filaments. Specifically, we found that mitochondria not only moved faster than Golgi stacks or peroxisomes in leaf epidermal cells of *N. benthamiana*, but they also maintained their directional movement for longer periods and covered larger distances before switching to more random motions. By contrast, peroxisomes spent very little time moving along linear paths and these linear motions were slower and did not last as long as those of mitochondria or Golgi stacks, possibly because of fewer myosin motors associated with peroxisomes. These results suggest that the movements of different organelles can be regulated independently of each other. At face value, this conclusion is most readily compatible with the direct-active model of organelle motility, which posits that movements depend on motors associated with the surface of individual organelles (Geitmann and Nebenführ, 2015).

Interestingly, our movement analysis also revealed that organelles spent, on average, only 2–7% of time in linear motion whereas 50–60% of the time was spent in wiggling motions. This finding implies that the organelle movements we have observed do not represent an indiscriminate ‘streaming’ motion but probably are tightly regulated.

ER movements are qualitatively different from those of organelles that form discrete spots as their speeds vary continuously along the network, and different regions of the network have vastly different mobility (Quader and Schnepf, 1986; Runions et al., 2006; Sparkes et al., 2009b). Although spheroid organelles also show great variation in speed, we were able to analyze specifically the faster linear movements for those organelles, whereas ER speeds are based on all regions of the network. A direct comparison of the speeds of these types of organelles is therefore not possible. Nevertheless, our quantitative approach allows us to detect relative changes and trends that reveal the interdependency of organelle movements in plant cells.

### Booster and Anchor constructs can alter the movement patterns of organelles in predictable ways

An important goal of this research was the creation of novel experimental approaches to specifically alter the movement of certain types of organelles. We reasoned that the recruitment of (additional) myosin motors to the surface of organelles should allow them to more readily engage with actin filaments and exhibit more processive and faster movements. Similarly, we expected that we could disrupt processive movement of organelles by binding them to cortical microtubules underneath the plasma membrane. Our experiments with transient expression of these Booster and Anchor constructs on mitochondria, peroxisomes and the ER validated these expectations and demonstrated that these approaches are able to alter the movements of the targeted organelles in a predictable way (Table S1). Addition of myosin-binding proteins to organelles resulted in faster linear movements of all organelles tested. In addition, Booster constructs resulted in longer runs and increased the net displacement of peroxisomes, suggesting that the additional myosin XI motors increased the processivity of peroxisome movements, as has previously been reported for *in vitro* experiments of myosin V and kinesin I (Nelson et al., 2014; Vershinin et al., 2007). Interestingly, this increased processivity was not detected for mitochondria, possibly because untreated mitochondria already recruit larger number of myosin motors. Alternatively, the high speeds of boosted mitochondria might have made it difficult to detect longer runs as they left the observation area or dipped out of focus. Unexpectedly, our findings of both higher speed and increased processivity are contrary to what was reported for kinesin motors on lipid droplets in *Drosophila* embryos, where no such changes were detected (Shubeita et al., 2008). The reason for this discrepancy is not clear, but the presence of multiple motors on the organelle might compensate for inefficiencies of individual myosin motors (Krementsova et al., 2017) as plant myosin XI motors might not be able to achieve processive movement on their own due to a low duty ratio (Haraguchi et al., 2018).

Interestingly, undirected wiggling motions could in some cases also be accelerated, suggesting that seemingly random motions do not necessarily reflect free Brownian motion but are at least partially driven by myosin motors (Brangwynne et al., 2009; Guo et al., 2014).

In contrast to the increased movements of organelles decorated with Booster constructs, the Anchor constructs increased the non-directed, wiggling motions and pause lengths, and reduced the speeds as well as run lengths of directed movements. These results indicate that Anchor constructs make directed motions of organelles more difficult and effectively fix individual organelles in place, which is also reflected in the reduced wiggling speed of anchored peroxisomes.

Interestingly, we found a lateral sorting of Anchor constructs within the ER membrane so that some parts of the ER network had increased persistency while other ER elements, particularly the cisternae, continued with their movements. A similar, although not as pronounced, lateral segregation within the ER membrane was found for Booster constructs, suggesting that feedback between membrane motility and membrane composition can lead to spontaneous patterning of the ER membrane into more static and more mobile sections.

### Organelles can move independently of others, but rapid movements can affect other organelles

A crucial question for research into cytoplasmic streaming is whether movement of organelles occurs independently of others by recruiting myosin motors to the surface of individual organelles, or whether there are indirect or collective behaviors that result in coupling of motility behaviors between different types of organelles (Geitmann and Nebenführ, 2015). Our finding that Anchor constructs had, by and large, no effect on the movement of non-targeted organelles (Table S1) suggests that these other organelles can move independently of the targeted organelles. This conclusion is consistent with the direct-active motility model that assumes motor binding to the surfaces of moving organelles, although we cannot exclude other interpretations. For example, it is possible that we simply did not target the Anchor constructs to the correct organelle that exerts the strongest indirect effect. Alternatively, the lateral segregation of the ER-Anchor might still allow sufficient ER movement in the interior of the cell to drive movement of other organelles. This interpretation could explain the difference between our findings and those described by Stefano et al. who found that globally slower ER speeds in *rhd3* mutants correlated with a significant drop in the average speed of mitochondria, peroxisomes and Golgi stacks (Stefano et al., 2014).

Unlike the experiments with Anchor constructs, the expression of some Booster constructs showed a clear effect on the motility of non-targeted organelles (Table S1). Unexpectedly, these indirect effects of Booster constructs depended on the targeted organelle. The mitochondrial Boosters had a marked effect on the three other organelles tested and stimulated linear movements of spheroid organelles as well as flow within the ER network. By contrast, peroxisome Boosters had mostly no effect on the movements of other organelles. It is conceivable that the much larger number of mitochondria is able to generate a hydrodynamic flow that secondarily facilitates the movement of other organelles, which would not be possible for the small number of peroxisomes. Our data on actin filament organization, however, suggests that the strong stimulatory effect of mitochondrial Boosters on other organelles might involve primarily an increased bundling of actin filaments. This increased bundling can be expected to favor faster and extended linear movements of all organelles (Akkerman et al., 2011). Our findings presented here complement the observed loss of prominent actin bundles in some myosin mutants with reduced organelle motility (Madison et al., 2015; Ueda et al., 2010). Importantly, we were able to induce increased bundling without overexpression of myosin genes, suggesting that these epidermal cells contain a pool of inactive myosin motors that can be recruited to organelle surfaces by providing additional binding sites. This opens the possibility that motor recruitment could be a major regulatory point for organelle motility.

Curiously, ER-Boosters had a stimulatory effect on Golgi stack and peroxisome movements, but not on mitochondria, demonstrating that the indirect effects on movements of non-targeted organelles can be specific for certain organelle types. This finding is consistent with the lack of increased actin bundling in ER-Booster-expressing cells and further suggests that ER movements do not cause a general hydrodynamic flow of the cytosol that passively drags other organelles along. It is tempting to speculate that the specificity of the ER-Booster effect is caused by direct interactions between ER membranes and Golgi stacks or peroxisomes. Indeed, several published studies suggest that there is a tight integration of ER exit sites and Golgi stacks in plant cells that frequently move together (daSilva et al., 2004). This binding appears to be quite strong as laser trapping of Golgi stacks can be used to extend ER tubules, an interaction mediated by the Golgi CASP protein (Osterrieder et al., 2017; Sparkes et al., 2009a). Similarly, peroxisomes are found in close association with ER membranes (Barton et al., 2013), although the protein(s) necessary for this association are not known. On the other hand, similar membrane contact sites have been described between ER and mitochondria, likely involving the MIRO2 protein on the outer mitochondrial membrane (White et al., 2020). It is not clear why these ER–mitochondrial contacts would behave differently from the ER–Golgi and ER–peroxisome contacts in our experiments.

### Anchor constructs induce morphological changes in mitochondria and the ER

Unexpectedly, we sometimes observed dramatic morphological changes of mitochondria and ER upon expression of their respective Anchor constructs which might affect their normal functioning. Mitochondria carrying the mito-Anchor constructs were on average larger and more elongated, suggesting that their attachment to microtubules affects their normal morphological homeostasis, possibly by stimulating fusion as previously suggested based on inhibitor treatments (Sheahan et al., 2005). It is also conceivable that tight association of mitochondria and cortical microtubules prevents normal fission of these organelles as this is thought to involve a constriction by proteins such as dynamin-related proteins and possibly the ER that wrap around the mitochondrion (Rose, 2021). Interestingly, elongated mitochondria have been previously observed in plant cells under conditions where the cellular energy level was low, such as after dark treatment or when oxygen is limited (Jaipargas et al., 2015), implying an adaptive mechanism that could optimize ATP production. On the other hand, we observed an apparent decrease in mitochondrial membrane potential as judged by reduced MitoTracker labeling suggesting reduced ability to generate ATP in mitochondria carrying the mito-Anchor constructs. Thus, the elongation of mitochondria induced by mito-Anchor constructs does not appear to employ the same mechanisms as those found in energy-depleted conditions and instead induces stress for the mitochondria.

The striking concentration of ER-Anchor constructs in a subset of ER tubules likely resulted from the free mobility of the proteins within the ER membrane combined with a trapping mechanism that retained the Anchors in cortical ER elements next to microtubules. This lateral segregation limited the effect of the ER-Anchor construct and allowed internal ER elements to move freely while the cortical tubules were locked in place. This effect differs from the normal observation that only small, local nodes of the ER are immobile (Griffing et al., 2014; Sparkes et al., 2009b).

Curiously, the binding of entire ER tubules to microtubules sometimes resulted in the bundling and rearrangement of microtubules into large, looped structures. Thus, our experiments with ER-Anchor proteins might have uncovered a feedback mechanism by which microtubules can recruit the ER to the cell surface but with the anchored ER at the same time dictating the direction in which microtubules are laid down. This rearrangement of microtubules can be expected to lead to changes in growth rate or direction of cell expansion, but the transient nature of our experiments did not allow us to test these predictions.

### Conclusion

In summary, we have generated novel engineering tools that enabled us to modify the motility of specific organelles in predictable ways. These experiments have revealed unexpectedly complex interactions between different organelles where movements of one type of organelle can affect those of other types of organelles. These indirect effects appear to be either specific for some organelles or general for all, suggesting different underlying mechanisms for these secondary impacts. In addition to the altered organelle motility, we also discovered changes in organelle morphology and even changes in cytoskeletal arrangements, which might have consequences for the normal functioning of the cell. Thus, the Booster and Anchor constructs described here provide a novel set of tools that can help to uncover powerful new insights into the dynamics of plant cells.

## MATERIALS AND METHODS

### Booster and Anchor constructs

Assembly of the Booster and Anchor constructs was achieved by conventional ligation of restriction digest fragments. The coding regions of transmembrane domains were obtained by reverse transcription PCR (RT-PCR) from total RNA of *Arabidopsis* Col-0 leaves with specific primers targeting TRANSLOCON OF THE OUTER MITOCHONDRIAL MEMBRANE7.1 (TOM7.1, At5g41685), PEROXIN12 (PEX12, At3g04460) and CALNEXIN1 (CNX1, At5g61790) (Table S2). The myosin-binding domain (DUF593, InterPro entry IPR007656) of MYOSIN BINDING PROTEIN1 (MYOB1, At1g08800) was also amplified from leaf cDNA, whereas the microtubule-binding domain (MBD) of mouse MAP4 was amplified from a plasmid containing a GFP–MBD fusion (Marc et al., 1998). The WAK2 signal peptide used in the ER-Booster and ER-Anchor construct was previously described (Nelson et al., 2007). The Booster and Anchor constructs were additionally tagged with a fluorescent protein, although the signal was often too low for reliable detection, particularly for the mitochondrial constructs. All Booster and Anchor constructs were cloned under control of the double 35S promoter and further inserted into the binary vector pPZP221 and transformed into *Agrobacterium tumefaciens* strain GV3101. Organelle markers used in this study were described previously (Nelson et al., 2007).

### *Nicotiana benthamiana* leaf infiltration

*Nicotiana benthamiana* plants were grown for 4–8 weeks before leaves were subjected to infiltration with *Agrobacterium tumefaciens* GV3101 carrying Boosters or Anchor constructs and various organelle markers following an established protocol (Batoko et al., 2000). Each construct and designated organelle markers were co-injected at an optical density at 600 nm (OD) of 0.05. At 48–72 h after infiltration, a small leaf region was excised and placed in a syringe filled with water to remove air from the mesophyll area by applying a light vacuum. Subsequently, leaf samples were placed on a glass-bottom dish with the abaxial side facing the coverslip and used immediately for imaging.

### Confocal microscopy

Leaf epidermal cells were imaged using a Leica SP8 white light laser confocal microscope with a 63×/1.4 NA oil immersion objective. Typically, images were collected with 8-bit bidirectional scanning with excitation at 514 nm for YFP, 569 nm for mCherry and 504 nm light for CFP. Zoom level was adjusted to 4.5 to achieve a pixel size of 80 nm. Scanning speeds of 1400 Hz line frequency (lines/second) was used to minimize delay between image frames.

### Epifluorescence microscopy

Spheroid organelles were imaged with a 100× (1.3NA) Plan-Neofluar oil-immersion objective on an Axiovert 200M microscope (Zeiss) equipped with a CCD camera (Orca ER; Hamamatsu Photonics) using filter sets for CFP, YFP and mCherry observation (filter sets 52017 and 69308, Chroma). Background images were obtained under the same settings and subtracted from the original images. Openlab software (Perkin Elmer) was used to capture timelapse videos with a time interval of less than 200 ms.

### Image analysis with TrackMate

Movement of punctate organelles was analyzed with TrackMate (Tinevez et al., 2017) in FIJI software (Schindelin et al., 2012) using an established pipeline (Nebenführ, 2020). Briefly, images were converted to 8-bit and background subtracted with a rolling-ball radius of 10 μm. Within TrackMate, object sizes were defined from 0.6 μm to 1 μm to identify mitochondria, Golgi stacks and peroxisomes. Thresholds were adjusted to minimize capturing non-specific signals. In order to identify as many organelles as possible, no other limitations were applied. The maximum allowed linking distances were based a maximal speed of 10 μm/s. Information from the spots and track statistics were saved as .txt and .tif images for subsequent analysis with custom macros that removed linking misassignments and classified movement steps into ‘linear’, ‘wiggling’ and ‘intermediate’. Specifically, we analyzed the ratio of net displacement over total displacement during four consecutive time intervals (Fig. S8). Movements with a ratio of ≥0.95 were labeled ‘linear’ and movements with a ratio of ≤0.5 were labeled ‘wiggling’. Ratios between 0.5 and 0.95 were labeled ‘intermediate’ and not analyzed further. For details, see Nebenführ (2020). Consecutive linear steps were treated as a ‘run’ and duration and net displacement were recorded. Similarly, consecutive wiggling steps were labeled ‘pauses’. We also recorded the speed of linear and wiggling steps separately.

### Analysis of ER movements

All ER images were acquired by confocal imaging and analyzed with AnalyzER software, which provides various speed parameters alongside with rigorous structure analysis for tubules and cisternae (Pain et al., 2019). Specifically, the program separates the ER network into tubules and cisternae based on the width of the fluorescent signal and reports average speed of movements along the ER network at different points within the structure (scalar sum, ‘local’), average speed for the entire structure (vector sum, ‘global’), and maximal speed. Here, we report only the upper quartile of global speeds of tubules and cisternae for which the flow coherence (mean local speed divided by mean global speed) is greater than 0.95 to ensure that random fluctuations are excluded.

### Shape analysis of mitochondria

YFP mitochondrial markers were expressed with and without the mito-Anchor in *Nicotiana benthamiana* epithermal cells as described above. Images were captured with epifluorescence microscopy, background-subtracted and thresholded. Circularity was calculated for all objects above the threshold with the ‘Analyze Particles’ particles in FIJI and ‘shape descriptors’ enabled.

### MitoTracker staining and analysis

After infiltrating *N. benthamiana* with mt-yb mitochondria marker, 500 nM MitoTracker Red CMxRos (Invitrogen) diluted in water was infiltrated into the previously infiltrated area. After 30 mins incubation, the stained leaf area was excised and subjected to confocal image acquisition after air removal. For MitoTracker detection, excitation at 579 nm and emission from 585 to 610 nm were used. Mitochondria-YFP signals were excited at 514 nm and emission captured from 520 nm to 540 nm. The background of both channels was subtracted with the rolling ball algorithm. Regions of interest (ROIs) regions were selected based on the YFP mitochondria images for intensity analysis. The same ROIs were applied to the MitoTracker images to calculate the ratio between the YFP and red channels.

### Colocalization analysis

Colocalization of organelles with microtubules was performed on confocal images with CFP organelle markers (with or without the corresponding Anchor construct) in *N. benthamiana* plants that stably expressed the α-tubulin fusion mCherry–TUA5 microtubule marker (Smertenko et al., 2020). Images were smoothed in FIJI software and processed with the JaCoP plugin (Bolte and Cordelieres, 2006). We report Pearson's correlation coefficient for an ROI enclosing the imaged cell after Costes thresholding. The Mann–Whitney test was performed in Prism (version 10).

### Actin filament imaging and analysis

Actin filaments were labeled with YFP–FABD2 (Park and Nebenführ, 2013; Voigt et al., 2005) in *N. benthamiana* epidermal cells transiently expressing the different Booster or Anchor constructs. Confocal image stacks covering the outermost 3 μm of the cells were captured on a Lecia SP8, deconvolved and collapsed into a single plane by maximal intensity projection. The bundle parameter was calculated for skeletonized actin filaments following a published protocol (Akenuwa et al., 2024).

### Statistical analysis

For statistical analysis, data from at least 15 cells per treatment were entered into GraphPad Prism (version 10). In general, we are reporting the upper quartile values of speeds, run durations, pause durations and net displacements as these values better represent the highly asymmetric distribution of measurements and thus better illustrate the movement potential of the observed cells. All treatment data were compared to the same set of control cells with the Kruskal–Wallis test with Dunn correction for multiple tests. To compare relative time spent in the movement categories, we used the Chi-square test in Excel (CHISQ.TEST).

## Supplementary Material

10.1242/joces.264573_sup1 Supplementary information
