# Supplementary material for: Accelerated organelle movements can indirectly affect the motility of other organelles in plant cells
Source: J Cell Sci. 2026 Sep 25;139(18):jcs264573. doi: 10.1242/jcs.264573 (PMC13680970; doi:10.1242/jcs.264573)
Supplement: Supplementary file 1 [file joces-139-264573-s1.pdf]

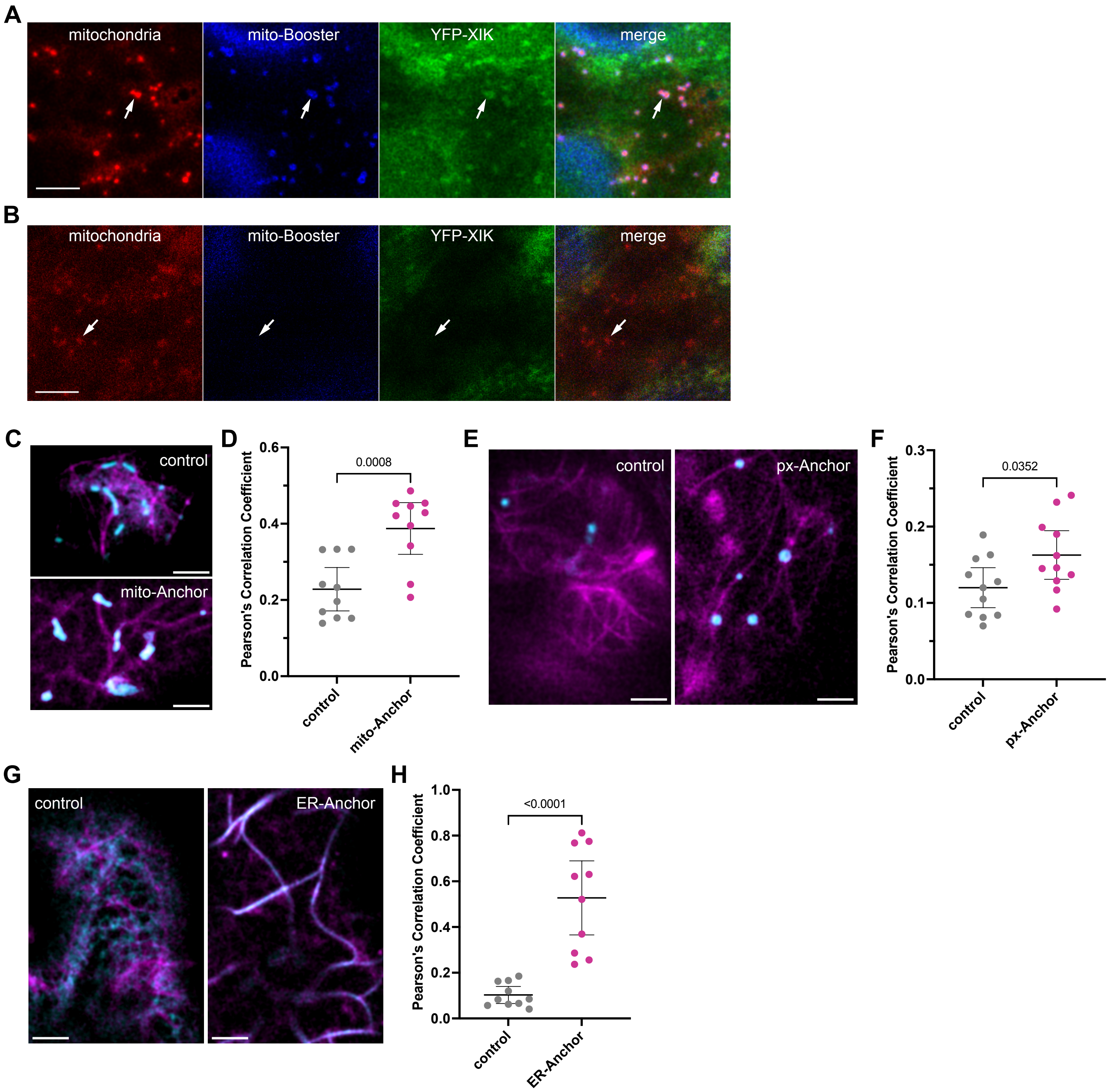

Fig. S1. Booster and Anchor constructs recruit myosin and microtubules, respectively. (A-B) Mito-Booster constructs recruit myosin motors to mitochondria. **(A)** Colocalization of a full-length myosin XIK fusion (YFP-XIK; green) to mitochondria (red) decorated with mito-Booster (blue). **(B)** In the absence of mito-Booster constructs, YFP-XIK does not localize to mitochondria (red). **(C-H)** Anchor constructs recruit their target organelles to microtubules. Target organelles were labeled with CFP (cyan) and microtubules with TUA5-mCherry (magenta). **(C)** Colocalization of mito-Anchor constructs with microtubules in control cells (upper panel) and cells expressing mito-Anchors (lower panel). **(D)** Quantitative analysis of mitochondria-CFP and mCherry signal overlap with Pearson's correlation coefficient. Every dot corresponds to one cell, horizontal lines indicate mean ± 95% confidence interval. Colocalization of mitochondrial and microtubule signal increased signifi-cantly with expression of the mito-Anchor construct (Mann-Whitney test). **(E)** Colocalization of peroxisomal and microtu-bule signal increased with expression of the px-Anchor construct (right panel). **(F)** Quantification of colocalization of peroxisomes and microtubules (Mann-Whitney test). **(G)** ER-Anchor constructs lead to stronger alignment of the ER with microtubules. **(H)** Colocalization of ER and microtubule signal increased with expression of the ER-Anchor construct ($p < 0.0001$, Mann-Whitney test). All scale bars are 5 μm.

Journal of Cell Science • Supplementary information

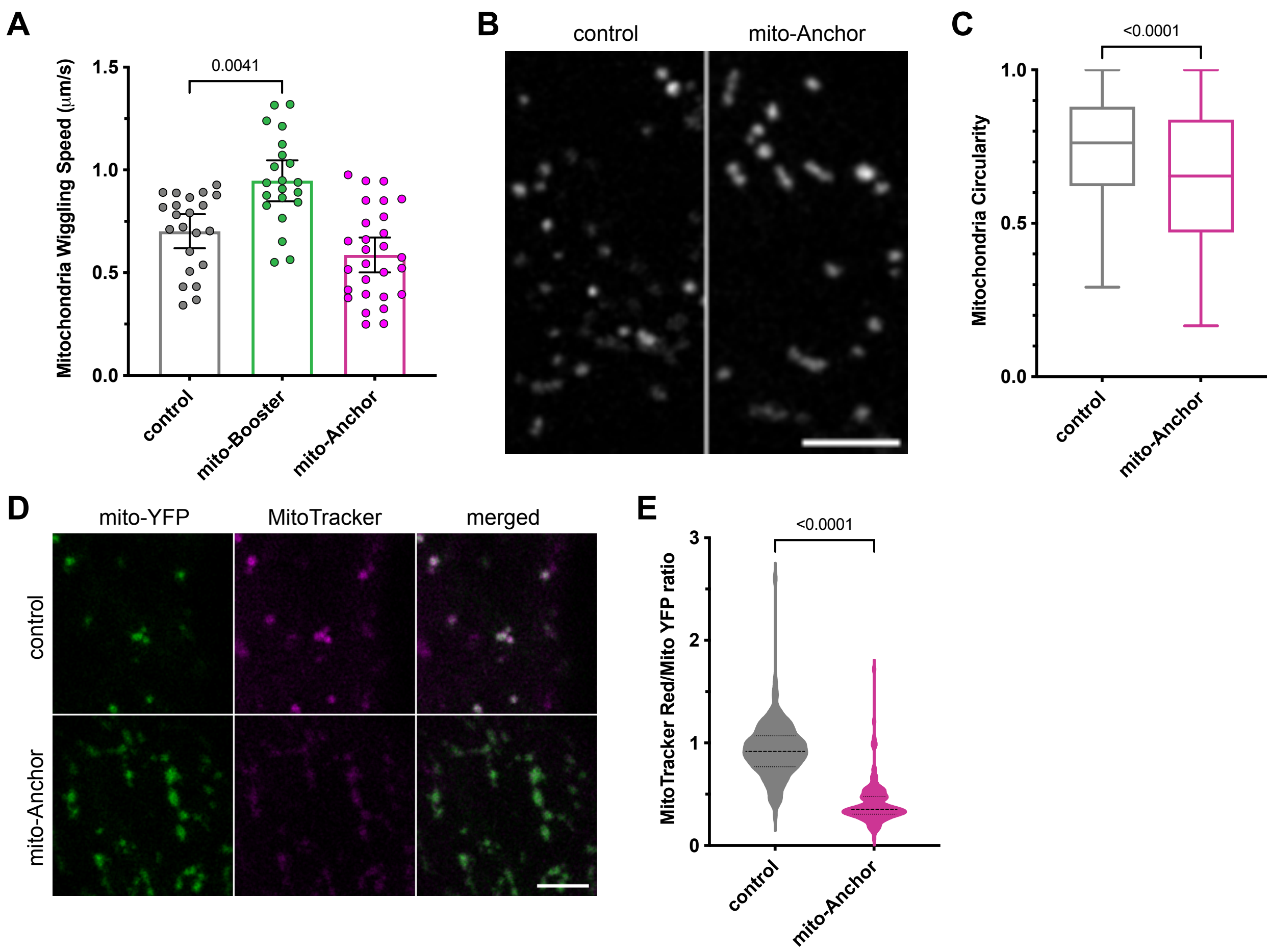

Fig. S2. Effects of mito-Anchor on mitochondrial shape and membrane potential.

(A) Wiggling speeds of mitochondria increased with expression of mito-Booster constructs. Every dot represents the upper quartile of instantaneous wiggling speeds measured in a single cell. Bar shows mean of all cells. Error bars represent 95% confidence interval. Significant p-values relative to control cells are indicated (Kruskal Wallis test with Dunn correction). **(B)** Mito-Anchors lead to an elongation of mitochondria. Representative images of mitochondria in control cells (left panel) and cells expressing mito-Anchors (right panel). Scale bar 5 μm. **(C)** Comparison of mitochondrial circularity in control cells and cells expressing mito-Anchors. Box plots show inter-quartile range, median as well as minimal and maximal values. Mann-Whitney test, n>170 mitochondria. **(D)** MitoTracker Red CMXRos labeling of mitochondria in control cells (upper row) and cells expressing mito-Anchor (lower row). Scale bar 5 μm. MitoTracker labeling of mitochondria was reduced in cells expressing mito-Anchor. **(E)** Violin plot showing distribution of ratio of MitoTracker Red and mito-YFP signal intensity; horizontal lines indicate median and upper and lower quartile. Mann-Whitney test, n>150 mitochondria.

Journal of Cell Science • Supplementary information

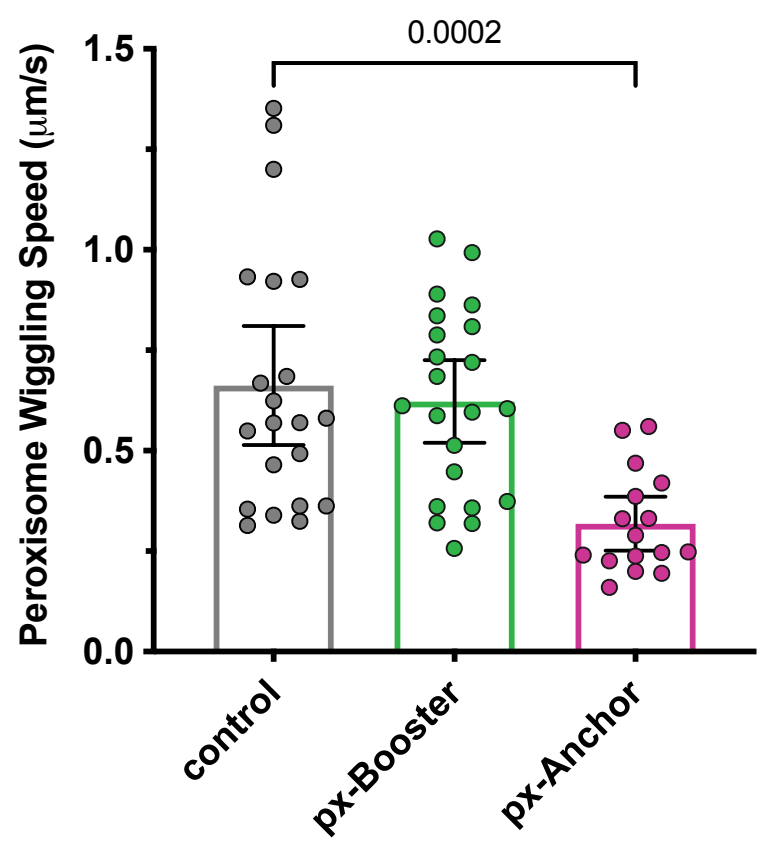

Fig. S3. Wiggling speeds of peroxisomes decreased with expression of px-Anchor constructs. Every dot represents the upper quartile of instantaneous wiggling speeds measured in a single cell. Bar shows mean of all cells. Error bars represent 95% confidence interval. Significant p-values relative to control cells are indicated (Kruskal Wallis test with Dunn correction).

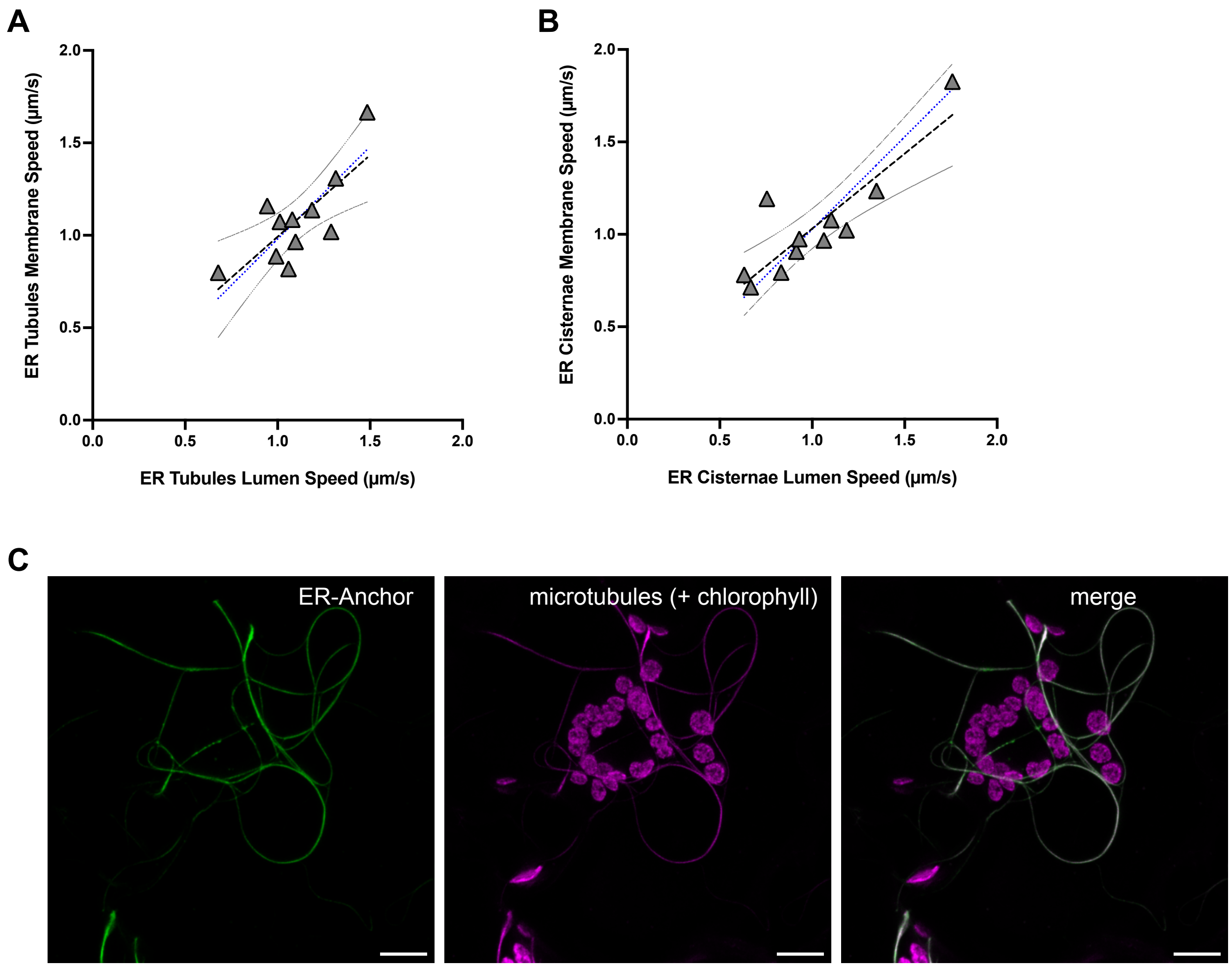

Fig. S4. Movement of ER lumen versus membrane; ER-Anchor effect on microtubules **(A,B)** Optical flow speeds of ER lumen marker and ER membrane marker are very similar. A soluble ER marker (CFP-HDEL) in the lumen of the ER (x axis) and a membrane-bound marker (YFP-CNX1) conformed to a 1:1 ratio (blue dotted line). Dashed black line shows linear regression of luminal and membrane speeds, with grey dotted curves indicating 95% confidence interval of this regression line. A Data for ER tubules. B Data for ER cisternae. **(C)** In some cells, ER-Anchor (green) expression caused dramatic rearrangement of microtubules labeled with TUA5-mCherry (magenta). Maximal intensity projection of a deconvolved confocal stack covering 31 μm. Scale bar 10 μm.

Journal of Cell Science • Supplementary information

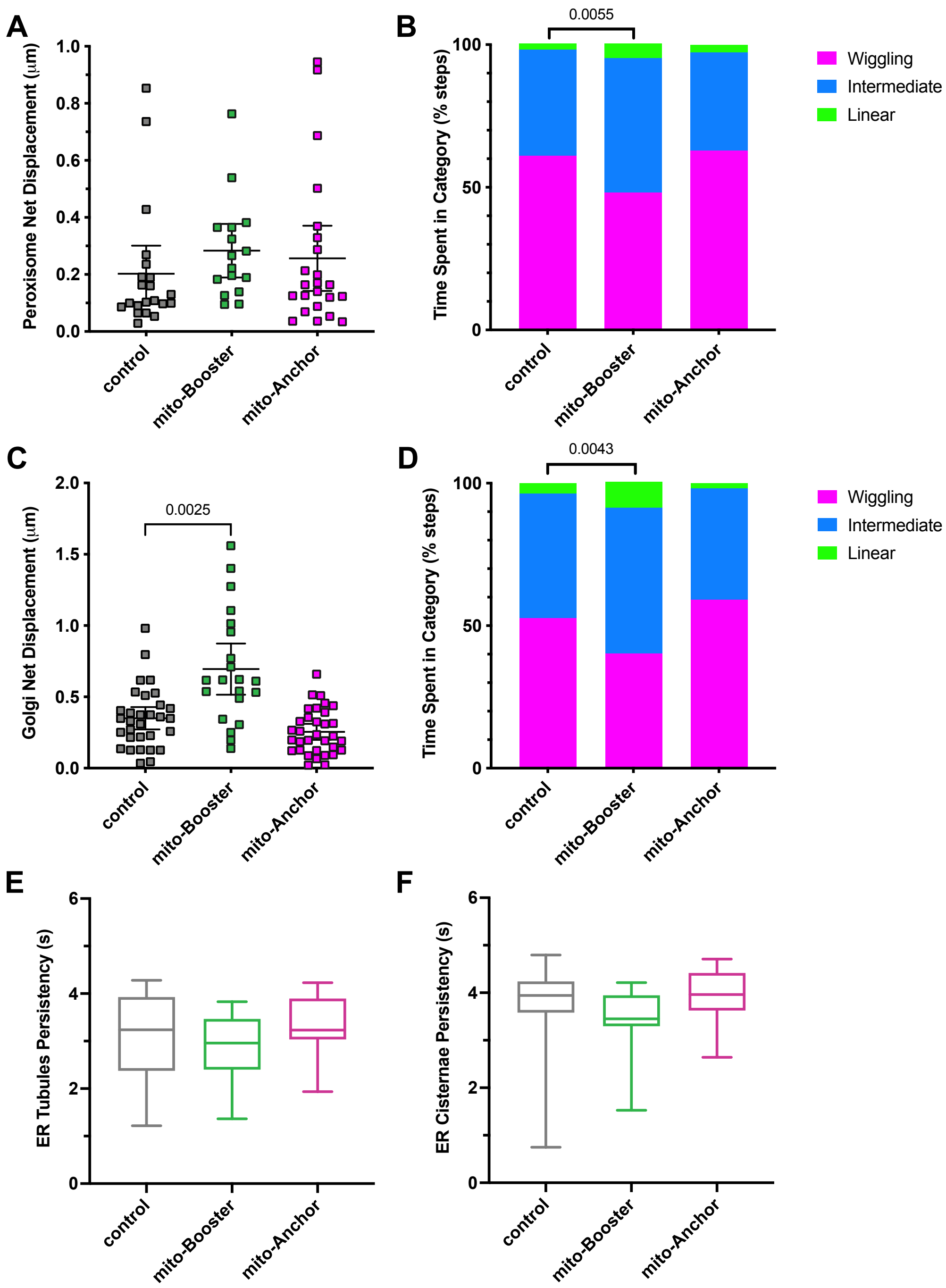

Fig. S5. Mitochondrial Booster and Anchor effects on other organelles

(A) Net displacement of peroxisomes during run episodes (upper quartile) were not significantly affected by mito-Boosters or mito-Anchors (Kruskal Wallis test with Dunn correction). **(B)** Mito-Booster expression increased the time peroxisome spent in linear motion and decreased the time spent in wiggling motion. Significant differences in proportions to control cells are indicated (chi-square test). **(C)** Net displacement of Golgi stacks during run episodes (upper quartile) increased significantly in with mito-Boosters expression. Significant p-values relative to control cells are indicated (Kruskal Wallis test with Dunn correction). **(D)** Mito-Booster expression increased the time Golgi stacks spent in linear motion and decreased the time spent in wiggling motion. Significant differences in proportions to control cells are indicated (chi-square test). **(E,F)** Persistency of ER tubules **(E)** and ER cisternae **(F)** did not change after expression of mito-Booster or mito-Anchor (Kruskal Wallis test with Dunn correction). Box plots show inter-quartile range, median as well as minimal and maximal values.

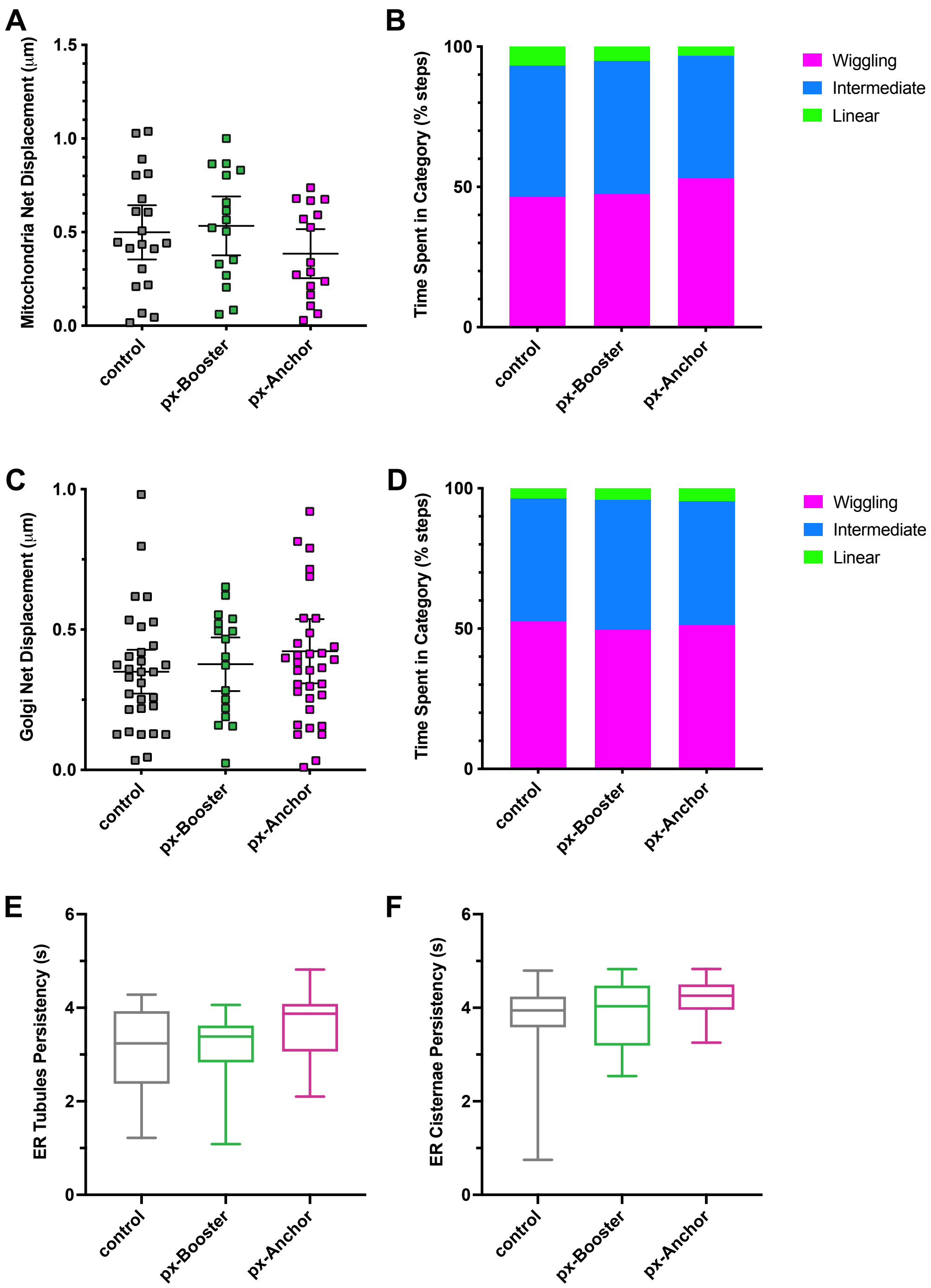

Fig. S6. Peroxisome Booster and Anchor effects on other organelles

(A) Net displacement of mitochondria during run episodes (upper quartile) were not significantly affected by px-Boosters or px-Anchors (Kruskal Wallis test with Dunn correction). **(B)** Px-Booster or px-Anchor expression did not affect the time mito-chondria spent in linear or wiggling motion (chi-square test). **(C)** Net displacement of Golgi stacks during run episodes (upper quartile) did not change significantly with either px-Booster or px-Anchor expression (Kruskal Wallis test with Dunn correction). **(D)** Px-Booster or px-Anchor expression did not change the time Golgi stacks spent in linear or wiggling motion (chi-square test). **(E,F)** Persistency of ER tubules **(E)** and ER cisternae **(F)** did not change after expression of px-Booster or px-Anchor (Kruskal Wallis test with Dunn correction).

Journal of Cell Science • Supplementary information

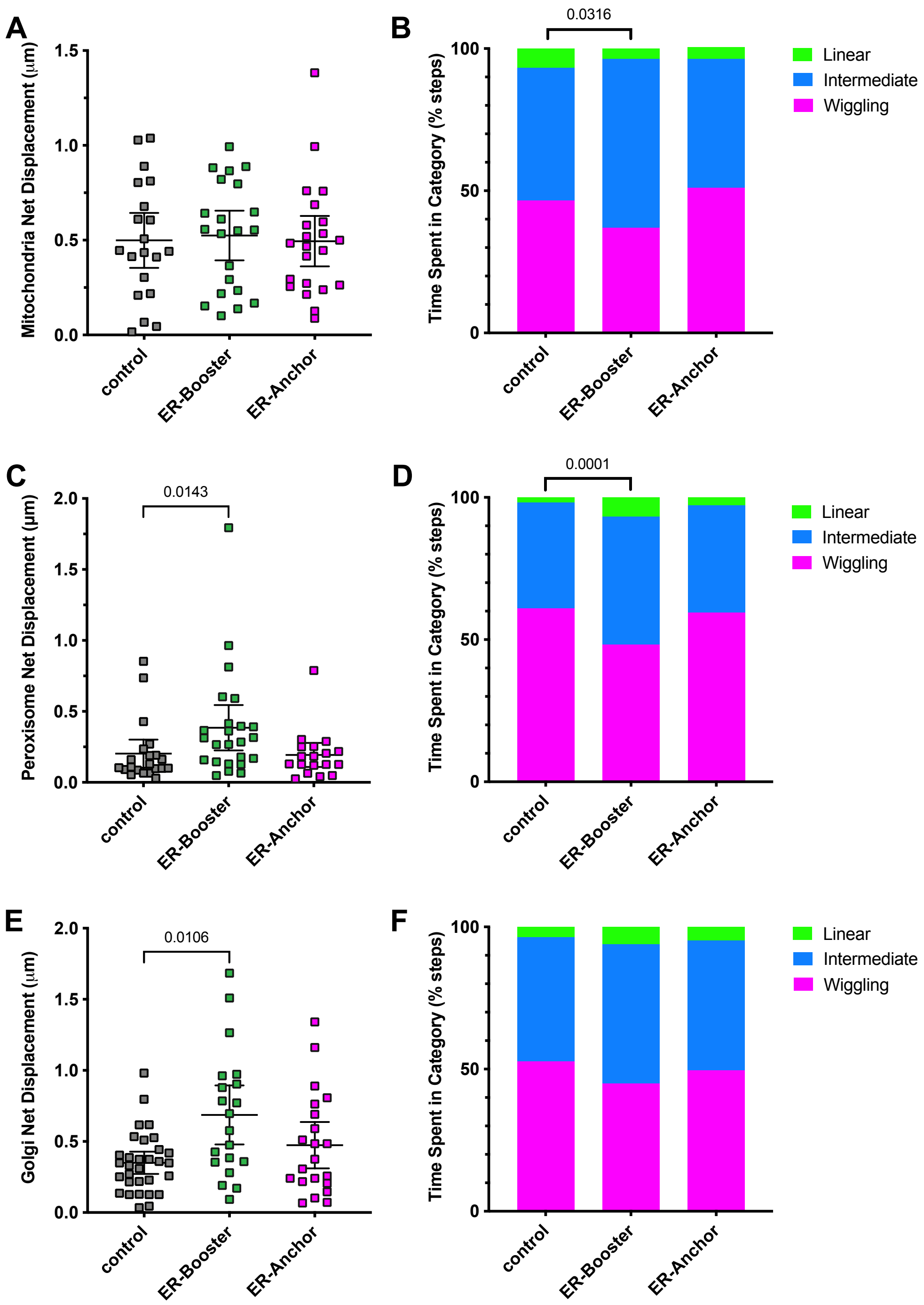

Fig. S7. ER Booster and Anchor effects on other organelles

(A) Net displacement of mitochondria during run episodes (upper quartile) were not significantly affected by ER-Boosters or ER-Anchors (Kruskal Wallis test with Dunn correction). **(B)** ER-Booster expression reduced the time mitochondria spent in linear or wiggling motion. Significant differences in proportions to control cells are indicated (chi-square test). **(C)** Net displacement of peroxisomes during run episodes (upper quartile) increased with ER-Booster expression (Kruskal Wallis test with Dunn correction). **(D)** ER-Booster expression increased the time peroxisomes spent in linear motion and reduced the time in wiggling motion. Significant differences in proportions to control cells are indicated (chi-square test). **(E)** Net displacement of Golgi stacks during run episodes (upper quartile) increased with ER-Booster expression (Kruskal Wallis test with Dunn correction). **(F)** ER-Booster or ER-Anchor expression did not change the time Golgi stacks spent in linear or wiggling motion (chi-square test).

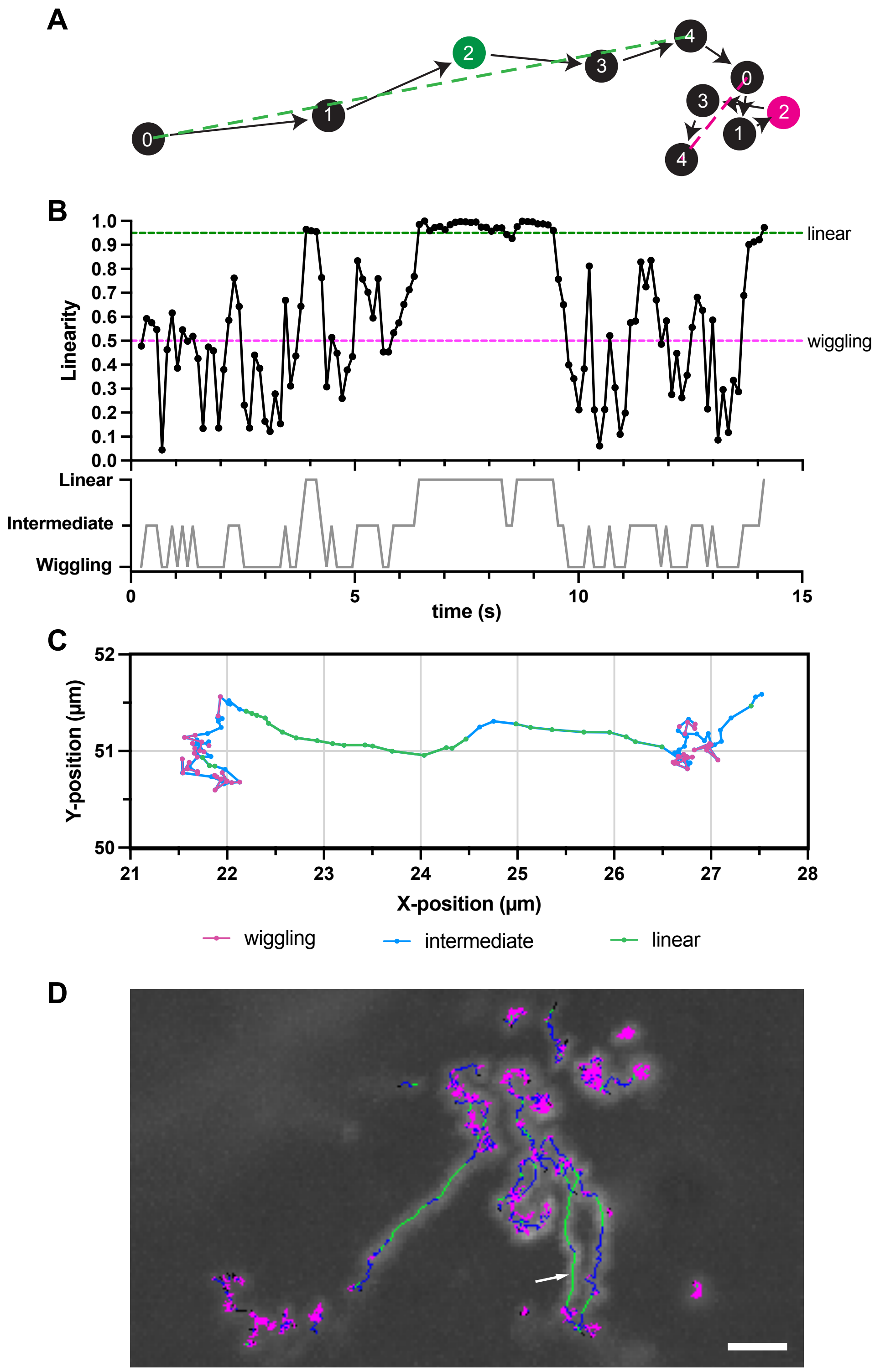

Fig. S8. Classification of movements in "linear", "intermediate" and "wiggling".
(A) For every time point (here labeled "2"), the four flanking time intervals (from point 0 to point 4) were used to calculate the ratio of net displacement (dashed colored line) to total displacement (sum of four displacements). Green spot shows a high ratio, or "linearity", magenta spot shows low linearity. **(B)** Change in linearity over time during Golgi stack movement. Time points with linearity > 0.95 were classified as linear movement, time points with linearity < 0.5 were classified as wiggling movement. Consecutive linear movements were labeled "Runs"; consecutive wiggling movements were labeled "Pauses." **(C)** Superimposition of spot classification on tracked Golgi stack from panel B. Time intervals between positions was 115 ms. Overall movement was from left to right. "Intermediate" labels (blue) indicate linearity values between 0.5 and 0.95. **(D)** Output of automated ImageJ macro with Golgi stack movement tracks color coded as in panel C. Arrow indicates movement track from panel C (rotated clockwise by 90°). Size bar = 2 µm.

Journal of Cell Science • Supplementary information

Table S1. Summary of changes in movement parameters by Booster and Anchor constructs.

Entries indicate the fold change over control conditions. Statistically significant differences to control conditions are color coded with green representing faster movement or increased processivity, and orange representing slower speeds or reduced processivity.

**MITOCHONDRIA**	**control**	**mito-Booster**	**px-Booster**	**ER-Booster**	**mito-Anchor**	**px-Anchor**	**ER-Anchor**
linear speed	2.05 µm/s	1.60	1.25	0.95	0.22	1.01	0.92
net displacement	0.50 µm	1.52	1.07	1.05	0.15	0.77	0.99
run duration	0.40 s	0.98	0.74	0.83	0.38	0.67	0.93
pause duration	0.88 s	0.85	0.80	1.10	1.56	0.90	1.17
% time linear	6.74	1.18	0.77	0.54	0.04	0.50	0.54
% time wiggling	46.62	0.81	1.02	0.79	1.54	1.14	1.09

**PEROXISOMES**	**control**	**mito-Booster**	**px-Booster**	**ER-Booster**	**mito-Anchor**	**px-Anchor**	**ER-Anchor**
linear speed	0.92 µm/s	1.23	2.62	1.45	1.12	0.42	1.05
net displacement	0.20 µm	1.40	4.44	1.90	1.27	0.33	0.95
run duration	0.24 s	1.16	2.72	1.63	1.07	0.80	1.00
pause duration	0.85 s	0.74	0.81	0.95	1.14	1.43	0.93
% time linear	1.77	2.69	12.42	3.81	1.61	0.39	1.61
% time wiggling	60.98	0.79	0.40	0.79	1.03	1.25	0.98

**GOLGI STACKS**	**control**	**mito-Booster**	**px-Booster**	**ER-Booster**	**mito-Anchor**	**px-Anchor**	**ER-Anchor**
linear speed	1.47 µm/s	1.23	1.19	1.67	1.02	1.28	1.00
net displacement	0.35 µm	1.40	1.08	1.96	0.73	1.21	1.35
run duration	0.33 s	1.16	0.94	1.36	0.74	1.00	1.29
pause duration	1.02 s	0.74	0.77	0.79	0.99	0.83	1.03
% time linear	3.67	2.69	1.13	1.67	0.49	1.25	1.30
% time wiggling	52.68	0.79	0.94	0.85	1.12	0.97	0.94

**ER**	**control**	**mito-Booster**	**px-Booster**	**ER-Booster**	**mito-Anchor**	**px-Anchor**	**ER-Anchor**
tubule speed	1.06 µm/s	1.22	0.95	1.51	1.04	0.88	0.89
cisternae speed	1.05 µm/s	1.37	1.04	1.68	1.05	0.92	0.93
tubule persistence	3.10 s	0.93	1.00	0.75	1.09	1.15	1.20
cisternae persistence	3.82 s	0.91	1.01	0.92	1.04	1.10	1.19

Table S2. Primers used.

Underlined sequences show restriction enzyme recognition sites used for cloning.

Name	Sequence	Product size
TOM7.1-N-F	CGA<u>AGATCT</u>GAGTCTACAATATCGTTGAAGG	
TOM7.1-stop-R	GCA<u>GCGGCCGC</u>ATGGATCAGACAGGGCTAAG	250 bp
PEX12-F1	CGA<u>ACTAGT</u>ATCACTAACTAGAAGAAGAGA	
PEX12-R1	GCA<u>GGATCC</u>AGTGTCCTGAAACAACCTCC	1237 bp
CNX-F1	CGA<u>TGTACA</u>GCGGATCCGATGTTGAGAAAGAGAAACAAAA	
CNX-R1	GCA<u>GCGGCCGC</u>CTATCCGGATGCAGATCTATTATCACGTCTCGGTTGCCTTT	420 bp
MyoB1-DUF-F	CGA<u>ACTAGT</u>ATG<u>AGATCT</u>TCGTTGGAAGGAGTTTCTGTG	
MyoB1-DUF-R	GCA<u>TCCGGA</u>CATTCCCTCACTAGGGGAAT	438 bp
MBD-F	CGA<u>ACTAGT</u>ATG<u>AGATCT</u>TCCCGGCAAGAAGAAGCAAA	
MBD-R	GCA<u>TCCGGA</u>ACCTCCTGCAGGAAAGTGG	1281 bp

Journal of Cell Science • Supplementary information

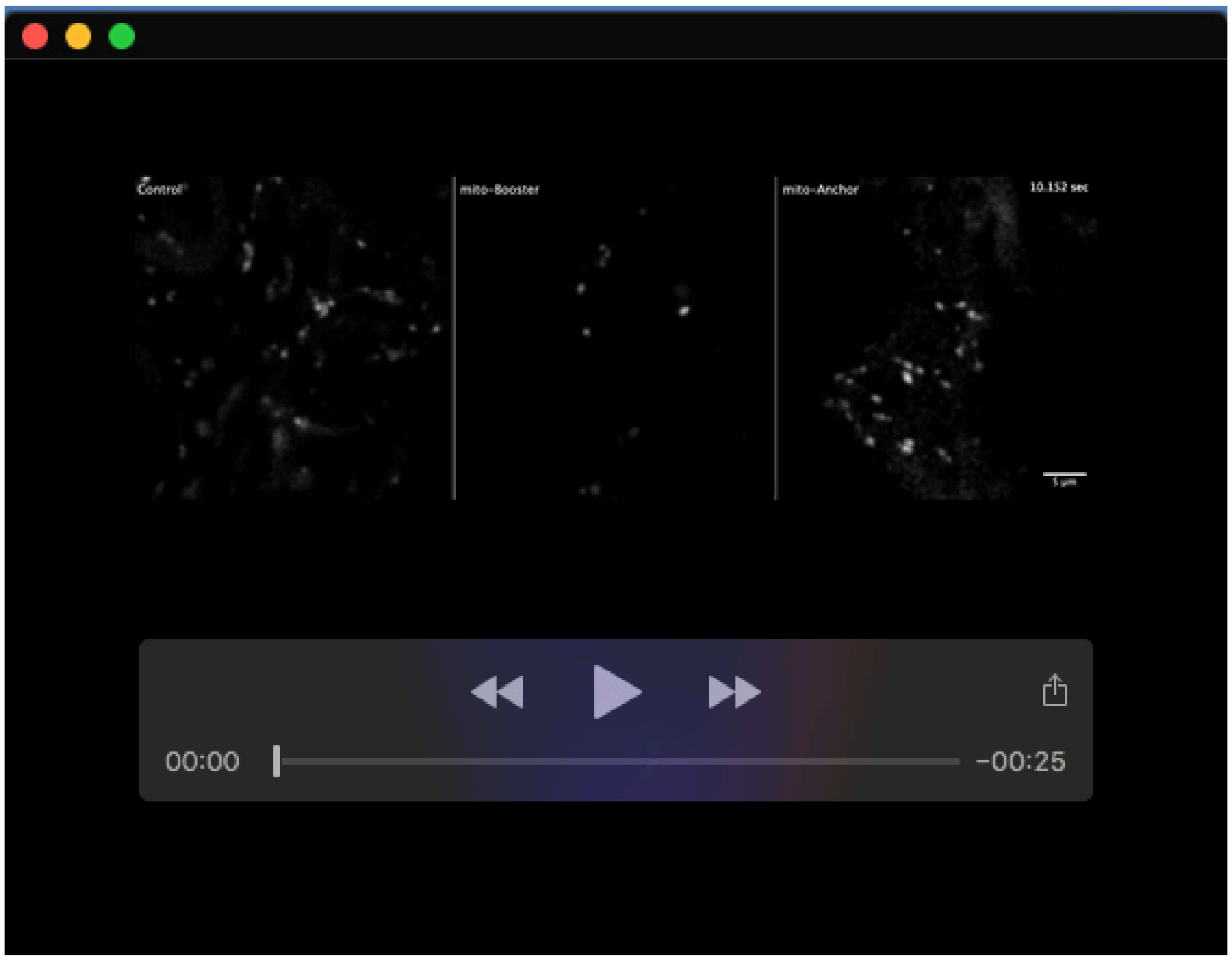

Movie 1. Movement of mitochondria in *N. benthamiana* leaf epidermal cells. Left panel: control cell; middle panel: cell expressing mito-Booster; right panel: cell expressing mito-Anchor. Confocal images were captured at 5.3 frames per second (Δt = 0.188s). Playback is at 16 frames per second, or three-times normal speed.

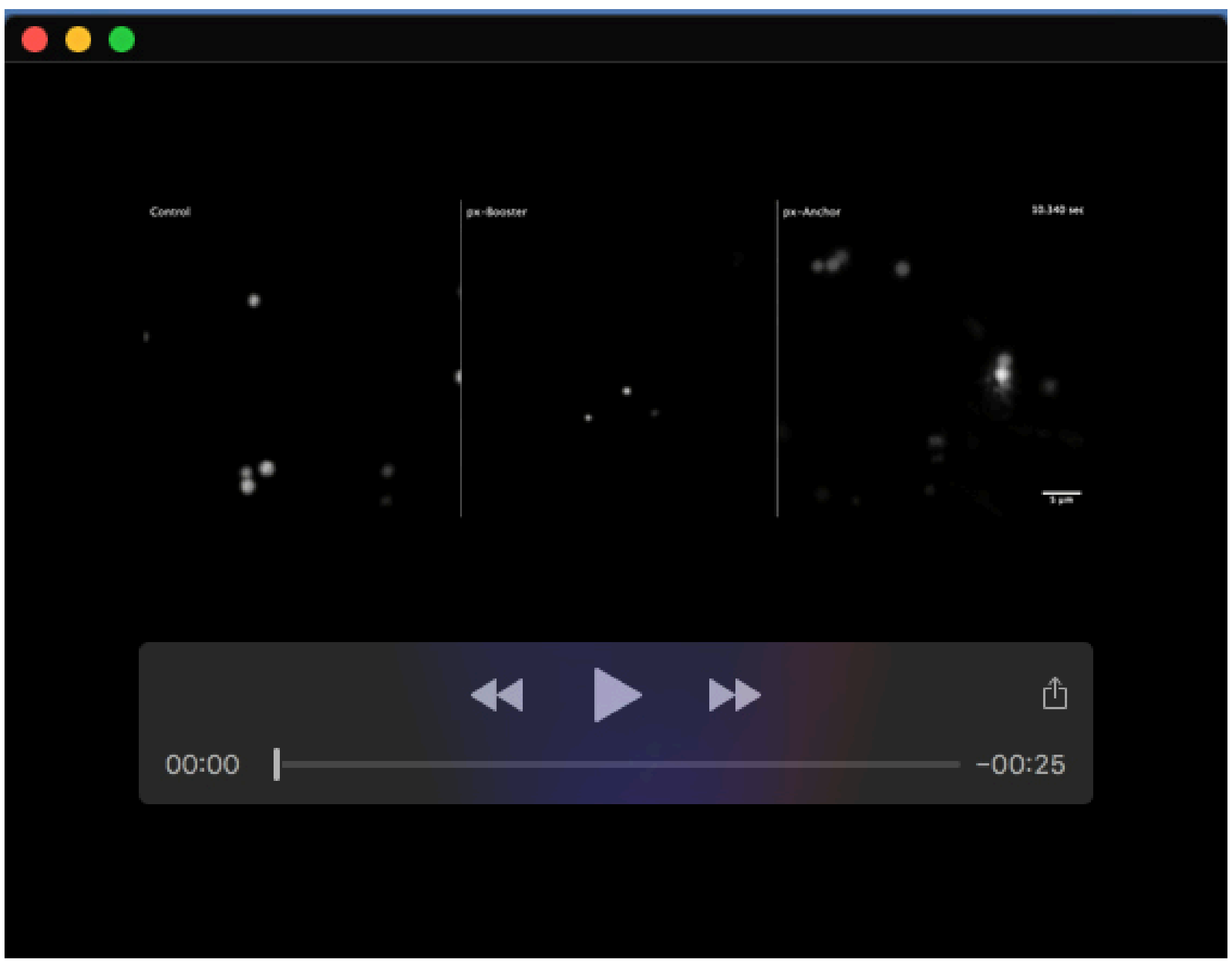

Movie 2. Movement of peroxisomes in *N. benthamiana* leaf epidermal cells. Left panel: control cell; middle panel: cell expressing px-Booster; right panel: cell expressing px-Anchor. Confocal images were captured at 5.3 frames per second (Δt = 0.188s). Playback is at 16 frames per second, or three-times normal speed.

Journal of Cell Science • Supplementary information

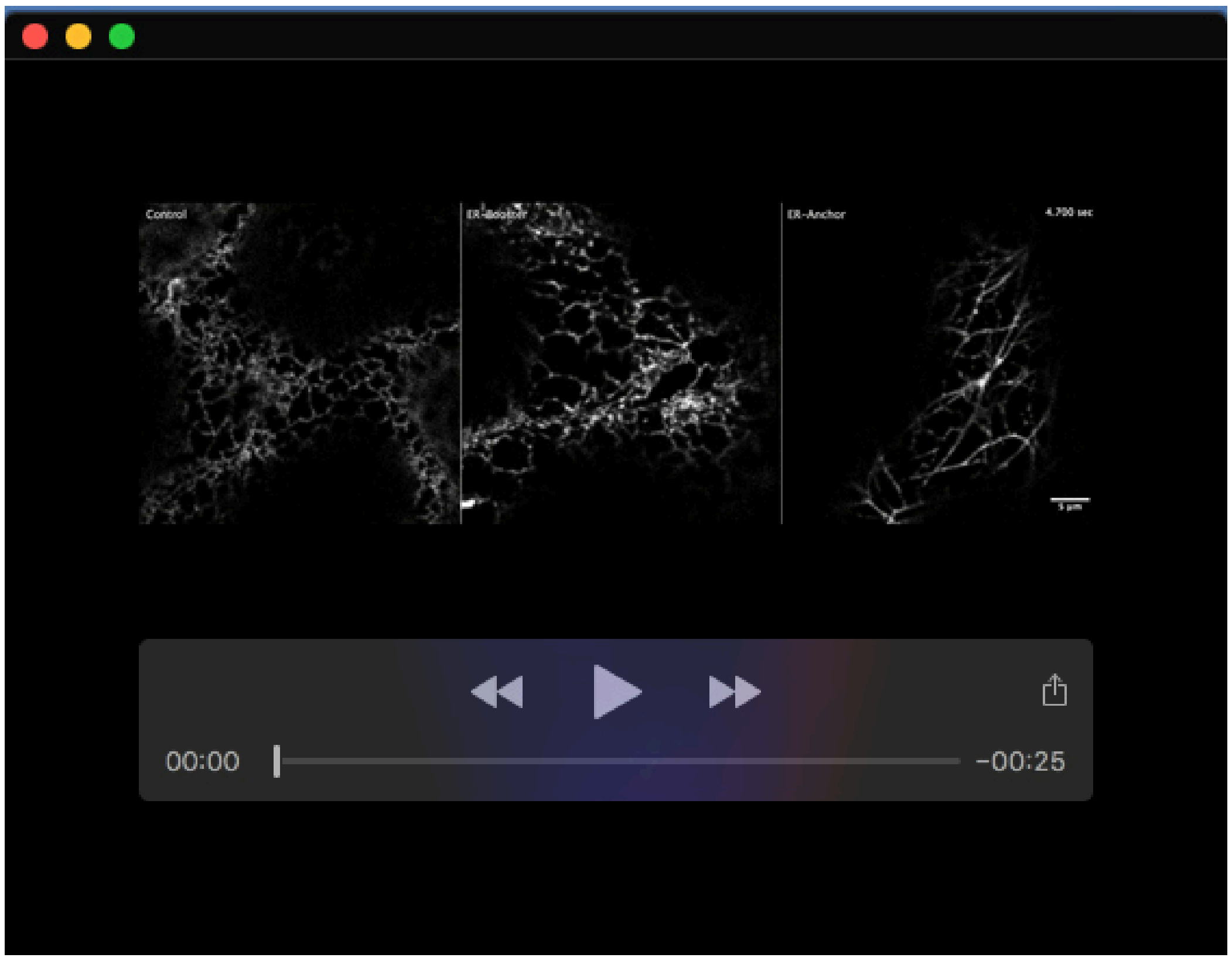

Movie 3. Movement of ER in *N. benthamiana* leaf epidermal cells. Left panel: control cell; middle panel: cell expressing ER-Booster; right panel: cell expressing ER-Anchor. Confocal images were captured at 5.3 frames per second (Δt = 0.188s). Playback is at 16 frames per second, or three-times normal speed.

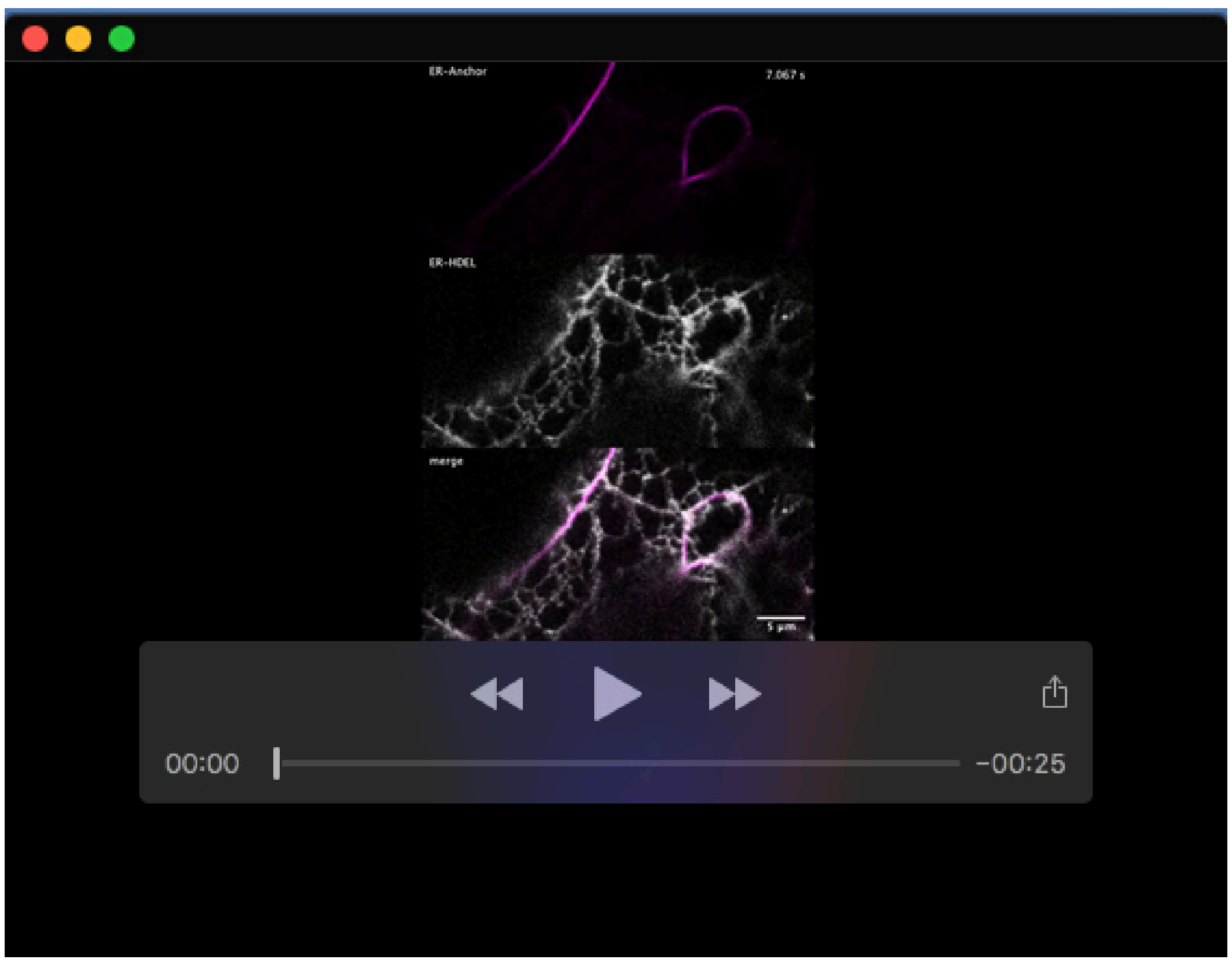

Movie 4. Movement of ER in *N. benthamiana* leaf epidermal cells expressing ER-Anchor. Upper panel: ER-Anchor signal (YFP), false colored magenta; middle panel: ER-HDEL marker (CFP); bottom panel: merged images. Confocal images were captured at 5.2 frames per second (Δt = 0.191s). Playback is at 15.7 frames per second, or three-times normal speed.
